# CRISPR: A Useful Genetic Feature to Follow Vaginal Carriage of Group B *Streptococcus*

**DOI:** 10.3389/fmicb.2017.01981

**Published:** 2017-10-11

**Authors:** Clémence Beauruelle, Adeline Pastuszka, Philippe Horvath, Franck Perrotin, Laurent Mereghetti, Philippe Lanotte

**Affiliations:** ^1^Université François Rabelais de Tours, UMR1282 Infectiologie et Santé Publique, Tours, France; ^2^INRA, UMR1282 Infectiologie et Santé Publique, Nouzilly, France; ^3^Service de Bactériologie-Virologie, Centre Hospitalier Universitaire de Tours, Tours, France; ^4^DuPont Nutrition and Health, Dangé-Saint-Romain, France; ^5^Inserm U930, Université François Rabelais de Tours, Tours, France; ^6^Département d’Obstétrique de Gynécologie et de Médecine Fœtale, Centre Hospitalier Universitaire de Tours, Tours, France

**Keywords:** CRISPR, *Streptococcus agalactiae*, carriage, genotyping, diversity

## Abstract

Clustered regularly interspaced short palindromic repeats (CRISPR) and Cas (CRISPR-associated proteins) play a critical role in adaptive immunity against mobile genetic elements, especially phages, through their ability to acquire novel spacer sequences. Polarized spacer acquisition results in spacer polymorphism and temporal organization of CRISPR loci, making them attractive epidemiological markers. Group B *Streptococcus* (GBS), a genital commensal for 10 to 30% of healthy women and a major neonatal pathogen, possesses a ubiquitous and functional CRISPR1 locus. Our aim was to assess the CRISPR1 locus as an epidemiological marker to follow vaginal carriage of GBS in women. This study also allowed us to observe the evolution of the CRISPR1 locus in response to probable phage infection occurring *in vivo*. We followed carriage of GBS among 100 women over an 11-year period, with a median duration of approximately 2 years. The CRISPR1 locus was highly conserved over time. The isolates that show the same CRISPR1 genotype were collected from 83% of women. There was an agreement between CRISPR genotyping and other typing methods [MLVA (multilocus variable number of tandem repeat Analysis) and MLST (multilocus sequence typing)] for 94% of the cases. The CRISPR1 locus of the isolates from 18 women showed modifications, four of which acquired polarized spacer, highlighting the *in vivo* functionality of the system. The novel spacer of one isolate had sequence similarity with phage, suggesting that phage infection occurred during carriage. These findings improve our understanding of CRISPR-Cas evolution in GBS and provide a glimpse of host-phage dynamics *in vivo*.

## Introduction

*Streptococcus agalactiae*, or Group B *Streptococcus* (GBS), is a major pathogen in humans and is the leading cause of neonatal infections in industrialized countries (Stoll et al., 2011; Edmond et al., 2012). Early-onset disease (EOD) (days 0–6) is the result of vertical transmission from a colonized mother during, or just before, delivery (Colbourn and Gilbert, 2007), whereas the pathogenesis of late-onset disease (LOD) (days 7–89) is less well understood (Berardi et al., 2013). GBS belongs to the commensal microbiota that colonize the gastrointestinal and genital tracts of 10–30% of healthy humans and can cause serious infections in neonates (Regan et al., 1991; Yancey et al., 1994). It is now universal practice to screen pregnant women for vaginal and/or rectal colonization with GBS to prevent early-onset infection (ANAES, 2001; Verani et al., 2010; Di Renzo et al., 2015). The aim of this screening strategy is to limit bacterial transmission and prevent EOD by the administration of intrapartum antibiotic prophylaxis. The GBS colonization status can change over the course of pregnancy. Thus, colonization status late in the third trimester has been used as a proxy for intrapartum colonization (at 35–37 weeks of gestation) (Anthony et al., 1978; Lewin and Amstey, 1981; Dillon et al., 1982; Hansen et al., 2004; Verani et al., 2010). In France, GBS screening is performed by swabbing the lower vagina followed by cultivation. This screening strategy must be repeated for each pregnancy, with the exception of a few special cases (women with GBS isolated from the urine during the current pregnancy or who had a previous infant with invasive GBS disease) (ANAES, 2001; Verani et al., 2010; Di Renzo et al., 2015). Moreover, screening for pathogenic bacteria, including GBS, must be performed in cases of imminent delivery risk (suspicion of chorioamnionitis, prolonged membrane rupture, or preterm labor) (ANAES, 2001).

Several methods have been developed to improve the diagnostic and prognostic classification of GBS isolates, such as serotyping, based on capsule polysaccharides, or, more recently, multilocus sequence typing (MLST), based on the sequencing of seven genes, which is expensive and time consuming (Jones et al., 2003; Lindahl et al., 2005; Slotved et al., 2007). These methods have highlighted the involvement of serotype III and sequence-type ST17 in causing more invasive neonate disease (Manning et al., 2009). Other molecular typing methods have been recently developed, such as multilocus variable number of tandem repeat (VNTR) analysis (MLVA), a molecular typing method based on VNTR variability (Haguenoer et al., 2011). This typing method targets six dispersed chromosomal loci. For GBS, it was shown to be more discriminant than MLST, especially for ST17 isolates (Haguenoer et al., 2011). CRISPR (clustered regularly interspaced short palindromic repeats) loci have also been described in GBS and used to genotypically characterize GBS isolates (Lopez-Sanchez et al., 2012; Lier et al., 2015).

Clustered regularly interspaced short palindromic repeats (CRISPR) and CRISPR-associated (Cas) proteins form the CRISPR-Cas system, which protects bacteria against bacteriophages (phages), and more generally, against exogenic mobile genetic elements (MGEs) (Barrangou et al., 2007). This system provides adaptive immunity through specific immunization based on nucleic acid sequences. CRISPR arrays are composed of short (25–40 bp) direct repeats (DR) interspaced by non-repetitive similar-sized sequences called spacers. DRs are highly conserved within a given CRISPR array and are often partially palindromic, having the potential to form hairpin structures (Kunin et al., 2007). Most CRISPR arrays are flanked on one side by an AT-rich sequence, called the leader, and on the other side by a trailer-end sequence, which is located downstream of the terminal direct repeat (TDR), a DR which is often degenerate and/or truncated. The CRISPR-Cas system acts in three stages (Barrangou and Marraffini, 2014). In the adaptation stage, a new spacer, usually derived from a foreign nucleic acid, is incorporated at the leader end of the CRISPR locus, concomitantly to the duplication of the leader-end DR. The expression stage consists on CRISPR RNA (crRNA) biogenesis, starting with transcription of the CRISPR array from a promoter located within the leader, followed by cleavage within the DRs of this long pre-crRNA molecule into short, mature crRNAs. Finally, during the interference stage, proteins target complementary foreign nucleic acids for cleavage within the sequence corresponding to the spacers. Thus, the CRISPR-Cas system provides sequence-specific immunity, whereby specificity is dictated by the sequence of the spacers that have been integrated within the CRISPR array. CRISPR loci thus show a dynamic, rapidly evolving, and polymorphic composition due to the ability of the system to capture novel spacers derived mostly from MGEs, reflecting past encounters with MGEs present in the environment. Spacer acquisition is polarized, as novel spacers are inserted at the leader end of the CRISPR locus. Consequently, sequences present at the trailer end can be considered to be ancestral, providing evidence of ancient MGE invasion events (Barrangou et al., 2007). Overall, spacer polymorphism and polarized acquisition of novel spacers result in the temporal organization of CRISPR loci, making them attractive epidemiological markers for genotyping and phylogenetic analyses (Shariat and Dudley, 2014).

Two CRISPR-Cas systems have been characterized in a collection of GBS strains (Lopez-Sanchez et al., 2012). A type II-A system, associated with the CRISPR1 locus, is ubiquitous and functional, whereas a type I-C system, associated with the CRISPR2 locus, is rare and most often incomplete, suggesting little or no activity (Lopez-Sanchez et al., 2012; Makarova et al., 2015). The CRISPR1 locus contains highly conserved 36-bp DRs separated by spacers of approximately 30 bp. Similarities between CRISPR1 spacer sequences and MGEs have been previously reported. Comparative sequence analysis of CRISPR1 loci across numerous strains revealed extensive diversity, due to the acquisition of new spacers, spacer duplications, and spacer deletions, illustrating the dynamics of the system (Lopez-Sanchez et al., 2012; Lier et al., 2015).

Previous studies reported the use of CRISPR1 spacer content and TDRs for the genotyping of GBS strains, showing a strong correlation between MLST and CRISPR-based genotyping (Lopez-Sanchez et al., 2012; Lier et al., 2015). Here, we used this genotyping method to follow vaginal carriage of GBS from 100 women with a minimum 3-month time interval between samples, and show that CRISPR1 polymorphism-based genotyping represents a rapid and discriminating tool for epidemiological studies. Furthermore, as spacer acquisition events occurred *in vivo* in multiple and independent cases during carriage, notably in response to a probable phage infection, our results strongly suggest that the CRISPR1-Cas system is functionally active for adaptation.

## Materials and Methods

### Bacterial Isolates

*Streptococcus agalactiae* isolates were collected between January 2004 and December 2014 in the Department of Obstetrics, Gynecology and Fetal Medicine of the CHRU of Tours, France, where approximately 4,000 deliveries are occurring each year. Isolates originated from gynecological (vaginal and endocervix) swabs, and for some patients, the gastric fluid of the neonate was kept as well. Isolates from gastric fluid aspirate samples originated from amniotic fluid and vaginal secretions ingested by the newborn during labor (Lanotte and Seme, 2012). The protocol was reviewed and approved by the Ethics Committee in Human Research of the University Hospital Center of Tours (approval number 2017-060) (Tours, France). Samples were considered in this study only if GBS isolates were obtained from the same women with a time interval of at least 3 months between two samplings. Among these women, 100 were randomly selected using the RAND function in Excel (Microsoft Office version 1997–2003). Isolates were stored at -80°C and grown on blood agar plates (TSH, BioMerieux^®^).

### DNA Extraction

Genomic DNA was extracted following enzymatic lysis with mutanolysin (Sigma). A bacterial suspension of 1.5 McFarland was prepared in 500 μL water containing 50 U of mutanolysin. The suspension was incubated for 1 h at 56°C, followed by 10 min at 100°C, leading to cell lysis. Lysates were centrifuged for 3 min at 1500 × *g* and the supernatants containing DNA collected.

### CRISPR1 Locus Sequencing

Clustered regularly interspaced short palindromic repeats1 locus amplification was performed in a T3000 thermocycler (Biometra) using CRISPR1 PCR-F and CRISPR1 PCR-R primers which target the CRISPR1-flanking regions, as previously described (Lopez-Sanchez et al., 2012). PCR amplification was performed in a total volume of 25 μL, containing 0.5 μM of each primer, 0.2 μM deoxynucleoside triphosphate (dNTP), 2 mM MgCl_2_, 0.02 U/μL Q5 high-fidelity DNA polymerase (New England Biolabs), 1× PCR buffer, and 5 μL extracted DNA. The PCR mixtures were heated at 98°C for 5 min, followed by 40 cycles of a denaturation step at 98°C for 30 s, an annealing step at 56°C for 30 s, an elongation step at 72°C for 120 s, and ending with a final extension step at 72°C for 10 min. PCR amplification was verified by electrophoretic migration in 1% agarose gel. PCR products were purified using centrifugal filter units (Millipore) as recommended by the manufacturer. The purified products were sequenced with BigDye Terminator Mix v3.1 on a Hitachi 3130xl Genetic Analyzer (Applied Biosystems), using the internal sequencing primers CRISPR1SEQ-F and CRISPR1SEQ-R, as previously described (Lopez-Sanchez et al., 2012). For CRISPR1 regions exceeding 1.3 kb, the use of primers targeting internal spacers was necessary to complete the sequencing of PCR products (Supplementary Table S1).

### CRISPR1 Sequence Analysis

DNA sequences were analyzed and assembled using the BioEdit sequence alignment editor version 7.2.5^1^. Spacers, repeats, and flanking regions for each sequence were identified using a macro-enabled Excel tool (P. Horvath, DuPont), which allows the identification and extraction of CRISPR features from nucleotide sequences, and the graphic representation of spacers as colored cells in Excel spreadsheets. Spacer sequences were compared to the dictionary of spacers established earlier (Lopez-Sanchez et al., 2012). New spacers identified in this study expanded the dictionary and were numbered incrementally. The spacer dictionary is available on the website^2^. Loci were labeled “identical” if all spacers were identical (same spacers in the same order) between two CRISPR1 loci. Two CRISPR1 loci were considered “close” if at least 70% of spacers were identical (same spacers in the same order), while they were considered “distinct” if at least 50% of spacers were different.

We performed a similarity search for each novel CRISPR1 spacer found in our isolates using BLAST^3^ with the Nucleotide collection (nr/nt) database and the default parameters of a prior analysis. All matches with a bit score above 40.0 and a query cover above 80% (corresponding to 100% identity) were retained. We performed a second search for spacer sequences without significant similarity using the whole genome shotgun contig (wgs) database limited to “*Streptococcaceae*” or “Firmicutes.”

### Multilocus Sequence Typing (MLST)

Multilocus sequence typing was carried out as previously described (Jones et al., 2003). Allelic profiles and sequence type (ST) were assigned using the international MLST database^4^. Clonal complexes (CC) were defined using the stringent group definition (6/7 shared alleles) and eBurst analysis^5^.

### Multilocus VNTR (Variable Number of Tandem Repeats) Analysis (MLVA)

In parallel, MLVA typing, targeting six loci, was carried out as previously described (Haguenoer et al., 2011). The number of repeats for each VNTR was deduced from amplicon size by electrophoretic migration in an agarose gel.

## Results

### Samples and Isolates Studied

**FIGURE 1:**
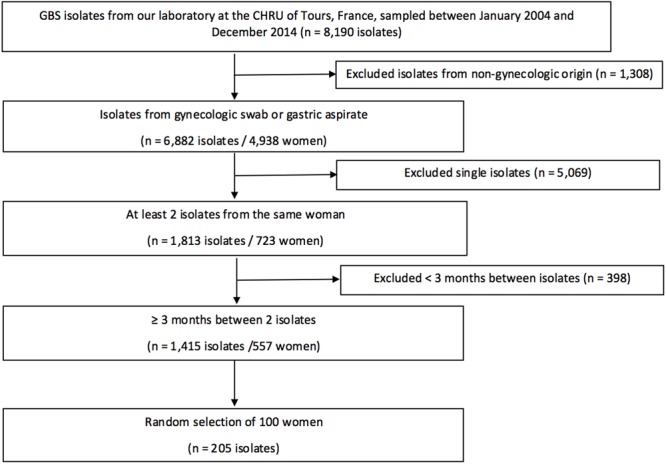
Study flow chart. Among the 8,190 isolates of our laboratory, multiple isolates originating from the same women were selected, and those from 100 women were further selected randomly to follow carriage evolution.

Among the 8,190 isolates of *S. agalactiae* collected between January 2004 and December 2014 at the CHRU of Tours, France, 205 isolates from 100 women were selected for the study (**Figure 1**). The average age of the cohort was 29.8 years at the time of sampling (median: 30 years, minimum: 19 years, maximum: 44 years). We analyzed two isolates for 95 women, and three for the remaining five. The isolates originated from vaginal swabs in 84% of cases (173/205), endocervical swabs in 11% (23/205), and gastric fluid samples from the neonate in 5% (9/205). The average time period between two samples was 2.4 years (median: 2.1 years, minimum: 3 months, maximum: 6.9 years). MLST analysis indicated that 51 isolates belong to CC1 (including 33 ST1), 46 to CC19 (including 24 ST19), 44 to CC23 (including 32 ST23), 26 to CC17 (including 24 ST17), and 21 to CC10 (including 5 ST10), whereas the remaining 17 isolates were ungrouped.

### CRISPR1 Locus Analysis

We obtained a complete CRISPR1 sequence for all isolates. The external primers (CRISPR1SEQ-F and CRISPR1SEQ-R) were sufficient for sequencing the entire locus for 123 isolates. Primers targeting internal spacer sequences were designed for the remaining 82. A total of 2,727 spacers were sequenced, including 240 not previously described in *S. agalactiae*. The average number of spacers per CRISPR1 locus was 13.8, with a minimal of three (isolates 43 and 44), and a maximal of 29 (isolates 24, 25, and 39). Detailed results obtained for each isolate are presented in Supplementary Figure S1.

**FIGURE 2:**
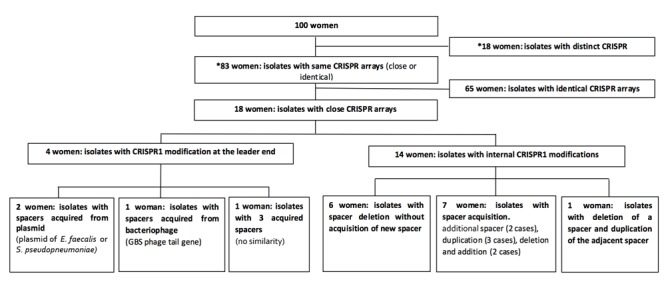
Diagram of case flow up to clustered regularly interspaced short palindromic repeats1 (CRISPR1) analysis. We compared CRISPR1 arrays of GBS isolated from the same women, and examined other regions of the genome by MLST and MLVA analysis to confirm the genetic relationships between isolates. ^∗^Three isolates from one women were collected, two with the same CRISPR1 locus, and one with a different one. The corresponding woman was thus classified into two groups (same CRISPR, and different CRISPR).

We compared CRISPR1 loci of isolates originating from the same women and observed “distinct” CRISPR1 loci between isolates for 18% (18/100) (**Figure 2** and Supplementary Figure S1A). GBS were isolated at three different dates for one of these women (number 98), and the last carried a different CRISPR1 locus than the first two. This woman was thus classified into both groups, “identical” and “distinct” CRISPR1 (**Figure 2**).

**FIGURE 3:**
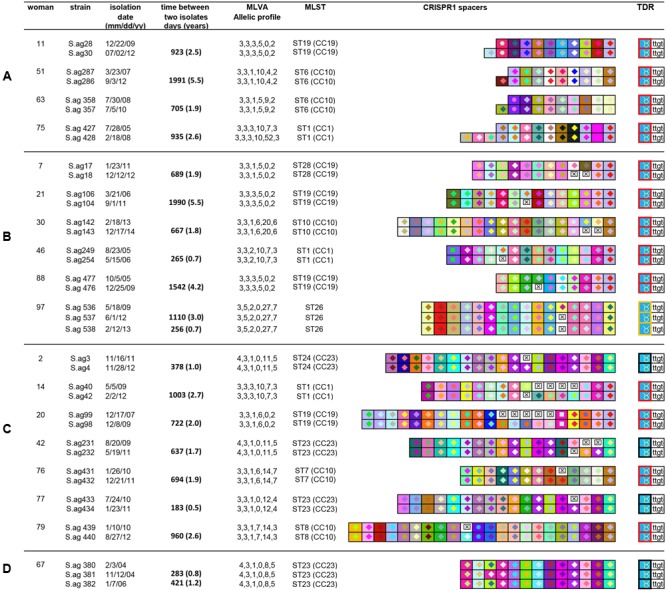
Comparison of the CRISPR1 arrays of isolates from 18 women where CRISPR1 modifications were observed. The CRISPR1 arrays are represented using a macro-enabled Excel tool, whereby spacers are converted into two-color symbols based on spacer sequence. Gaps (=missing spacers) are shown with a boxed cross symbol (⊠) after alignment of identical spacers between strains of the same group. The MLVA allelic profile corresponds to the number of repeats of each VNTR (SAG2, SAG3, SAG4, SAG7, SAG21, and SAG22) (Haguenoer et al., 2011). Terminal Direct Repeats (TDRs) are represented by different colored borders according to their sequence. **(A)** CRISPR1 arrays with leader-end acquisitions; **(B)** CRISPR1 arrays with internal deletions; **(C)** CRISPR1 arrays with internal additions; **(D)** CRISPR1 array with a spacer switch (women 67). The second of three isolates shows a spacer replacement, likely resulting from a duplication of the downstream spacer.

The isolates contained a “close” or “identical” CRISPR1 locus for 83% of the women (83/100) (Supplementary Figure S1B); the isolates of 65 women presented an identical CRISPR1 locus, whereas those for the other 18 contained “close” loci. We observed only slight variations in spacer composition, including additional spacers at the leader end and deletion or acquisition of internal repeat-spacer unit(s) (**Figure 3**). Spacer sequences are provided in Supplementary Figure S2.

The final isolate from four of these 18 women had one (women 11, 51, and 63) or three (woman 75) additional repeat-spacer units at the leader end of CRISPR1 compared to the older isolate (**Figure 3A**). There is no sequence similarity between these six novel spacers and those already described in GBS. In contrast, they share similarities with plasmid or phage sequences. The novel spacer of strain 30 (woman 11) is similar to an *Enterococcus faecalis* pLG2 plasmid sequence (identities: 30 nucleotides /30), whereas that of strain 286 (woman 51) is similar to a *S. pseudopneumoniae* pDRPIS7493 plasmid sequence (identities: 30 nucleotides /30). The spacer acquired by strain 357 (woman 63) is similar to a *S. agalactiae* phage tail gene (identities: 30 nucleotides /30), whereas the three novel spacers of strain 428 (woman 75) do not show any similarity with known sequences. The average time for the acquisition of these novel spacers was 3.1 years (median: 2.5 years, minimum: 1.9 years, maximum: 5.5 years) (**Table 1**).

**Table 1:** Period between sampling of isolates deriving from the same ancestor, among the 83 women with close or identical clustered regularly interspaced short palindromic repeats1 (CRISPR1) arrays.

	No CRISPR1 locus modification (65 women, 133 isolates)	Leader-end modification (4 women, 8 isolates)	Internal locus modification (14 women, 29 isolates)
Average (years)	2.0	3.1	2.3
Median (years)	1.8	2.5	1.9
Minimum (years)	0.3	1.9	0.5
Maximum (years)	5.6	5.5	5.5

We observed internal CRISPR1 locus modifications for isolates originating from 14 women. The final isolate for six of these women has deletions of repeat-spacer units: one-unit deletion in four isolates (women 21, 46, 88, and 97), and two-unit deletion in the two others (women 7 and 30) (**Figure 3B**). The final isolates originating from the seven other women present additional repeat-spacer units in internal regions of the CRISPR1 array: one unit in four cases (women 2, 76, 77, and 79), and two, four, or five units in the other cases (women 42, 14, and 20, respectively) (**Figure 3C**). In three cases, the additional spacers correspond to a single spacer duplication (women 2, 76, and 77). The duplicated spacer is the adjacent one in two cases (women 2 and 76), but a distant spacer in another case (woman 77). In two other cases, the additional spacers were not already present in the locus (women 20 and 79). In two cases (woman 14 and 42), internal spacer acquisition was seemingly combined with the deletion of an adjacent spacer. Spacer acquisition corresponded to the duplication of two spacers (woman 42) or the acquisition of four new spacers (woman 14). Interestingly, a spacer switch occurred in one case (woman 67) (**Figure 3D**). The intermediary (out of three) isolate originating from this woman shows a spacer replacement likely resulting from a duplication of the downstream spacer. As the initial and final isolates contain an identical CRISPR1 locus, it is likely that this strain was present, but not isolated, during the second sampling. The average time for internal modification of the CRISPR1 locus was 2.3 years (median: 1.9 years, minimum: 6 months, maximum: 5.5 years) (**Table 1**). Among the five isolates with spacer duplications (4, 232, 432, 434, and 381), four belong to CC23. Overall, we observed spacer duplication in the CRISPR1 locus for 73 isolates (35.6%). In 24 isolates, the duplication involved spacer 247 in our dictionary, which is CC23-specific and shares sequence similarity with *Streptococcus* phage phi3396. This spacer is present in 82% of CC23 isolates [36/44, including two isolates (293 and 295) where it was truncated], and duplicated in 54% of them (24/44) (Supplementary Figure S1).

We compared modifications of CRISPR1 loci in the isolates with their phylogenetic affiliations as established by MLST (**Table 2**). The polarized acquisition of novel spacer(s) at the leader end involved one isolate belonging to CC1 (ST1), one belonging to CC19 (ST19), and two belonging to CC10 (ST6). We observed no CRISPR1 locus modification for isolates belonging to CC17.

**Table 2:** Phylogenetic affiliation and CRISPR1 array modifications among isolates from 83 women with close or identical CRISPR1 arrays.

	CC1 (*n* = 19)	CC19 (*n* = 19)	CC23 (*n* = 17)	CC17 (*n* = 12)	CC10 (*n* = 9)	Others^∗^ (*n* = 7)	Total (*n* = 83)
No modification	16	14	13	12	4	6	65
Middle locus modification	2	4	4	–	3	1 (ST26)	14
Leader-end spacer acquisition	1 (ST1)	1 (ST19)	–	–	2 (ST6)	–	4

*^∗^ST22 (isolates from two women), ST130 (isolates from two women), ST26 (isolates from two women), ST243 (isolates from one woman).*

### MLST and MLVA Analysis

To confirm the genetic relatedness between isolates, phylogenetic markers dispersed throughout the GBS chromosome were analyzed using MLST and MLVA (**Table 3**). There was concordance between MLST analysis and CRISPR1 typing for 99% of cases (isolates from 99/100 women), whereas 94% of cases showed concordance between CRISPR1 typing and MLVA. We observed a differing CRISPR genotype for one woman (woman 70, isolates 396 and 397), but these isolates actually belong to the same ST (ST19) and have the same MLVA profile (Supplementary Figure S1A). The CRISPR1 locus from these isolates shows similarities at the level of ancestral spacers (trailer end) and TDR, but high variability across leader-adjacent spacers. With the exception of this case, we systematically observed different MLVA profiles (94.5%; 17/18 women) among women for whom isolates possess different CRISPR1 arrays. We observed the same MLVA profile for 94% of women (78/83) among those from whom isolates possess identical or “close” CRISPR1 arrays. In isolates of 6% of women (5/83), the MLVA analysis showed a different profile, due exclusively to a modification in the SAG21 VNTR (women 33, 34, 35, and 98 with isolates having identical CRISPR1 arrays, and woman 75 with isolates having “close” CRISPR1 arrays).

**Table 3:** Agreement between CRISPR genotyping and other typing methods (MLST and MLVA).

		CRISPR array	
		Different	Close or identical
MLVA	Different	17 (94.5%)	5 (6%)
	Identical	1 (5.5%)	78 (94%)
			
MLST	Different	17 (94.5%)	-
	Identical	1 (5.5%)	83 (100%)
			

Total		18	83

*There was an agreement between the three typing methods for 94% of women. For the others women, one presented isolates with a different CRISPR genotype but belonging to the same ST and have a same MLVA profile (woman 70). The other five women (33, 34, 35, 75, and 98) presented a same CRISPR genotype, a same ST but a different MLVA profile always due to SAG21 VNTR.*

### Analysis of CRISPR1-Flanking Regions and Internal DRs

Finally, we analyzed the flanking regions of CRISPR1 (leader and trailer ends), as well as internal DRs, as these sequences sometimes show variations between groups of strains. The CRISPR1 trailer sequence was conserved for all isolates except for ST22 isolates which shared five additional nucleotide following DRT (Supplementary Figure S1B). The CRISPR1 leader sequence was identical for 180 isolates (87.8%) over the 130 bp located immediately upstream of the first DR. Among the 27 isolates showing differences, the last nucleotide was deleted in 15 instances, or duplicated in one case (Supplementary Table S3). This last nucleotide modification concerned CC10 isolates in 87.5% of cases (14/16). For one isolate, 26 additional nucleotides were found at the 3′ end of the leader, corresponding to a partial DR sequence, likely originating from an incorrect tandem duplication of the first repeat. A single nucleotide difference was present in the leader sequence of the 10 remaining isolates. In contrast, DR sequence analysis across all CRISPR1 loci showed a high conservation of the internal DRs, with only six isolates showing SNPs among the 2,727 DRs (isolates 17, 43, 44, 231, 420, and 421). The TDR was represented by five different sequences, including four described previously (Supplementary Table S2) (Lier et al., 2015). TDR sequences were specific for each CC, except for CC1 and CC19 that share the same TDR sequence (Supplementary Figure S1).

## Discussion

In this work, we explored the *in vivo* dynamics of human colonization by GBS over several years by comparing the CRISPR1 loci of 205 isolates collected from 100 women over an 11-year period. The aims of this study were to evaluate CRISPR1 locus analysis as an epidemiological marker, to describe the evolution of CRISPR1 arrays in GBS over time during human carriage, and to assess the likelihood of phage infection *in vivo*, highlighted by alterations of the spacer content, notably additions at the leader end of CRISPR1.

Eighteen women (18/100) provided GBS isolates with a “close” CRISPR1 locus over the sampling time. MLST typing confirmed the genetic relationship between these isolates, as well as MLVA analysis, except for one case which differed only at the SAG21 VNTR locus (woman 75), as discussed below. These isolate pairs arguably share common ancestry, and the observed CRISPR locus modifications are the result of *in vivo* evolution within patients. Modifications at the leader end (four women) correspond to spacer acquisition which likely occurred in response to MGE invasion events. Two isolates show similarities between novel spacers and plasmids from related species (*E. faecalis* and *S. pseudopneumoniae*), consistent with previous studies showing similarity between most GBS spacers and streptococcal genome sequences (Lopez-Sanchez et al., 2012). One isolate acquired a novel spacer showing strong similarity with a gene encoding a phage tail protein, probably originating from a Caudovirales/*Siphoviridae* phage, as are most *Streptococcaceae* phages (Domelier et al., 2009). For one woman, the two isolates differed from each other by the polar acquisition of three spacers that show no similarity to any known sequence. These spacers could have been acquired successively, or all at the same time, during the 2.5 years separating the two samplings. Overall, polarized spacer acquisition at the leader end was relatively rare in our population. Indeed, this event occurred in only 4% of the colonized women of the study. The mean time between sampling of GBS isolates showing polarized acquisition was 3.1 years (compared to 2.3 years for all isolates). Metagenomic analyses of the population dynamics of MGE has shown a rapid ability of CRISPR-Cas systems to acquire spacers in both environmental and human niches (Fineran and Charpentier, 2012). In the *S. thermophilus* type II-A CRISPR system, just one *in vitro* challenge with a phage was sufficient to result in the acquisition of new spacers (Barrangou et al., 2013). Similarly, *in vitro* biofilm analysis showed a high rate of evolution for *Leptospirillum* type II CRISPR arrays, whereby the number of spacers increased from 37 to 197 in 5 months (Tyson and Banfield, 2007; Andersson and Banfield, 2008). Pride et al. (2011) analyzed *Streptococcus* spp. (*S. thermophilus, S. mutans, S. pyogenes*, and *S. agalactiae*) CRISPR loci directly from the saliva of four patients over 18 months by evaluating spacer number by PCR amplification with primers directed at common DRs. These authors showed for each patient a high rate of alteration of spacer number at one CRISPR locus, over time, demonstrating the dynamics of the CRISPR1 system in human. In our study, the rare cases of spacer acquisition might be explained by a low occurrence of MGEs in this environment, or by a low activity of the CRISPR1-Cas system in GBS. Furthermore, as no lytic phage has been isolated yet from GBS, the acquisition of new spacers showing similarity to phage sequences may correspond to adaptation from excised prophages.

In our study, we observed two types of modifications of the internal CRISPR1 array: spacer acquisitions, and deletions. Deletions always occurred in an internal region of the locus or close to the last spacer, as previously described (Deveau et al., 2008; Horvath et al., 2008; Lopez-Sanchez et al., 2012). These deletions never concerned spacers in contact with the leader or TDR. Deletions and spacer duplications may result from slipped misalignment during DNA replication (Lopez-Sanchez et al., 2012). For two isolates (women 20 and 79), the acquired spacers were not already present in the locus, and acquisition could be due to homologous recombination, which has been described in GBS (Bisharat et al., 2004; Brochet et al., 2008; Lopez-Sanchez et al., 2012). In two cases (women 14 and 42), internal spacer acquisition -including a spacer duplication case- was seemingly combined with the deletion of an adjacent spacer. A similar phenomenon probably also occurred for the intermediary isolate of woman 67, from whom three isolates were studied; the first and third isolates were identical, while the intermediate isolate differed by a duplication and deletion of one spacer.

We observed a correlation between GBS phylogenetic lineages and the ability to integrate new spacers in CRISPR1, suggesting an impact of the genetic background on adaptation efficiency. Isolates with polarized acquisition belong only to ST1, ST19, or ST6, whereas internal locus modifications were observed for isolates belonging to CC1, CC19, CC23, CC10, and ST26 (**Table 2**). We observed no CRISPR1 modification for CC17 isolates, neither in at the leader end nor in the middle of the locus, despite their relatively large representation (26 isolates from 18 women). Moreover, CC17 isolates showed less spacer variation at CRISPR1 locus, suggesting lower activity of CC17 CRISPR system. This result is consistent with previous observations which also highlight a significantly lower number of spacers in CC17 isolates in comparison with isolates belonging to other lineages (Lier et al., 2015). Nevertheless, Domelier et al. (2009) demonstrated the unique character of CC17 phages, in addition to a considerable prophage diversity in ST17 isolates. This is consistent with previous studies which compared inter-species CRISPR loci, reporting a strong inverse correlation between the number of spacers and prophages within a given genome (Nozawa et al., 2011). Similarly, CC23 isolates displayed less spacer variation, as previously described, and shared a relatively high number of common spacers (Lier et al., 2015). We found that internal spacer duplication was more frequent among CC23 isolates. Notably, spacer 247 was duplicated in 54% of CC23 isolates and was highly represented (82% of CC23 isolates). This spacer shares similarity with *S. dysgalactiae* phage *phi* 3396, which likely originated from a *S. pyogenes* phage (Davies et al., 2007).

Furthermore, we noticed an association between phylogenetic affiliation and leader sequence. The leader sequence is highly conserved in GBS, as well as in other bacteria (Yosef et al., 2012; Lier et al., 2015; Wei et al., 2015). Notably, the sequence at the leader-repeat junction is critical for adaptation by CRISPR-Cas systems, as shown in *Escherichia coli* type I-E (Yosef et al., 2012) and *S. thermophilus* type II-A (Wei et al., 2015). Among our isolates, the leader-repeat junction was modified in 8% of them (17/205), and isolates with leader-repeat junction modifications mostly belonged to CC10 (82%, 14/17). This modification was frequent and occurred in 67% of the CC10 isolates (14/21), in agreement with prior observations (Lier et al., 2015).

Our study showed a strong congruence between the various typing methods used, namely CRISPR typing, MLST, and MLVA. We explored other regions of the GBS genome by MLVA and MLST (exploring six and seven loci distributed throughout the chromosome, respectively) to confirm the relationship between the isolates. For one woman (woman 70), the two isolates carried different CRISPR1 loci but displayed the same MLST and MLVA profiles, suggesting a genetic link. Their CRISPR1 arrays contained five identical ancestral spacers and the same TDR, confirming common ancestry. However, spacers located near the leader showed high variability, likely due to an independent recent evolution. For these isolates, whole genomic analysis could be applied to evaluate their relationship.

For five patients, isolate pairs having identical or close CRISPR1 loci had the same ST but different MLVA profiles. Among them, the second isolate of one woman (woman 75) acquired three spacers at the leader end of CRISPR1. The only difference between MLVA profiles were found within the SAG21 VNTR and may be due to repeat sequence acquisition or deletion over time. Of note, SAG21 is the most variable VNTR among those used for MLVA (Haguenoer et al., 2011) and is perhaps too variable for MLVA typing for middle- to long-term carriage of GBS, and typing over a long period. The rate of evolution of this locus has not been explored yet.

These results need to be interpreted with caution. Although spacer content of the CRISPR1 locus is generally specific for isolates for a given woman, some isolates from different women (isolates from women 5 and 6, and those from women 68 and 18) and belonging to CC23 had identical CRISPR1 loci, as observed previously (Lier et al., 2015). Similarly, some isolates belonging to ST17 had close CRISPR1 loci, with a change in just the most recent spacer (isolates from women 99, 26, and 87). Identical CRISPR1 loci among strains of different origin and belonging to ST17 were observed previously by Lopez-Sanchez et al. (2012). This may be due to the circulation of highly prevalent clones of CC23 or CC17, even if there is no evidence of an epidemiological link between the different isolates, except pairwise.

Another aim of this study was to evaluate the evolution of GBS during carriage using these three typing methods. Among the 100 women studied, 78% presented carriage of GBS strains with the same profile, irrespective of the molecular method used (CRISPR1 typing, MLVA, or MLST), and 83% presented carriage of the same GBS strain with the same or close CRISPR1 array. Similar GBS carriage has been previously described (Hansen et al., 2004; Manning et al., 2008). Hansen et al. (2004) followed carriage for 21 months (during pregnancy and up to 1 year after delivery) by pulsed-field gel electrophoresis (PFGE). In their study of 35 carriers, 86% of women were colonized by just one clone, and the others by two clones simultaneously, and no clonal shift was observed (Hansen et al., 2004). Manning et al. (2008) explored perinatal GBS colonization (during pregnancy, and 6 weeks after delivery) by serotyping, GBS capsular gene cluster genotyping, and MLST. Comparison of the three typing methods revealed differences, with clonal replacement in 18% of women, and a change of ST for 10% (Manning et al., 2008). However, these longitudinal studies were performed during a short period (maximum 21 months between two samples). Our study, based on a longer period, extends previous findings on the predominance of one clone during carriage, using highly discriminant typing methods. One limitation of our study is that the stored isolates may represent the predominant strain in cases where two or more coexisted in the sample. This approach does not provide a complete overview of bacterial diversity over time and we cannot exclude the possibility of multiple populations, particularly if a strain was predominant. Indeed, carriage of a double population has been shown previously (Hansen et al., 2004; Perez-Ruiz et al., 2004; Brzychczy-Włoch et al., 2014). This double population may explain our observations for woman 67, where the second isolate carried a different CRISPR1 locus than that of the first and third isolates. The discriminatory power provided by CRISPR1 locus analysis could make this a simple and relatively rapid typing method to explore GBS vaginal carriage.

Clustered regularly interspaced short palindromic repeats-based typing provides a glimpse of GBS vaginal carriage at the molecular level. This method has the advantage of being linked to the genetic lineage of the isolate through analysis of the TDR and ancestral spacers. Furthermore, it allows exploration of the recent evolution of the isolate, especially encounters with MGEs, through analysis of the leader end. The CRISPR-based typing method appears to be a useful tool to compare GBS isolates, thanks to its high discriminatory power and ease of use. However, further studies are required before CRISPR can be used more generally as a typing method for GBS. In addition, it is now evident that the GBS CRISPR1 locus evolves *in vivo*. We observed polarized acquisitions in response to MGEs (plasmids or phages), highlighting the dynamics of the system. The ability of the CRISPR1 array to evolve may be linked to phylogenetic affiliation, exemplified by the apparently lower ability of the hypervirulent strain ST17 to acquire novel spacers, compared to the higher ability of ST6 isolates. This potential link is yet to be explored.

## Author Contributions

Conceived and designed the work: CB and PL. Performed manipulations: CB and AP. Drafted the paper: CB, PH, and PL. Critically revised the manuscript: PH, FP, and LM. All authors read and approved the final version of the manuscript.

## Conflict of Interest Statement

PH is an employee of DuPont, and co-inventor on several patent applications related to CRISPR-Cas systems. The other authors declare that the research was conducted in the absence of any commercial or financial relationship that could be construed as a potential conflict of interest.

## Footnotes

1 http://www.mbio.ncsu.edu/bioedit/bioedit.html

2 http://crispr.i2bc.paris-saclay.fr/CRISPRcompar/Dict/Dict.php

3 https://blast.ncbi.nlm.nih.gov/

4 http://pubmlst.org/sagalactiae/

5 http://eburst.mlst.net/v3/mlst_datasets/
