# Supplementary material for: CRISPR: A Useful Genetic Feature to Follow Vaginal Carriage of Group B Streptococcus
Source: Front Microbiol. 2017 Oct 11;8:1981. doi: 10.3389/fmicb.2017.01981 (PMC5641575; doi:10.3389/fmicb.2017.01981)
Supplement: Supplementary file 1 [file Data_Sheet_1.pdf]

Supplementary Material

CRISPR: a useful genetic feature to follow vaginal carriage of Group B *Streptococcus*.

Clémence Beauruelle, Adeline Pastuszka, Philippe Horvath, Franck Perrotin, Laurent Mereghetti and Philippe Lanotte *

*Corresponding author: philippe.lanotte@univ-tours.fr

Table S1. Internal primers used for CRISPR1 locus sequencing.

Primer name	Sequence (5' → 3')
Sp335F	AAGGGTGTTAGATGATAATACCTTTTTTAA
Sp2166F	TGTTTTTTCTTTCCATATGTGGTGGCTTTC
Sp856F	TATCAAGTCTGCTTTGTCAACAAAGTCAGC
Sp2234F	AGTTTGTTTATGATGAACACCAAAATTTAA
Sp325F	GGATGATTTCGATTATGCGGCGGTGGTTGA
Sp511F	AGTTACTTCTGCTTGGGTTTGATAAGGGTC
Sp40F	TATGTCTTCTAACAGTTGCTTCTTGTGCTT
Sp531F	AGTTTAGAGTTAACGTCTGTATTTTAGATA
Sp109F	ACAAAATCTTTTGTTGCTCCTGGACGTATT
Sp823F	AACACAGCTTCCTCGAAAGGGATATATCTA
Sp2069F	CCGTGCAAGAGTTAAGGAACTGACACAGCA
Sp2201F	GAAAAATTAAATACTTTCCAATAATTATAA
Sp2191F	AACCTAAAAAGATGTATGAGAAGTTGCCTT
Sp650F	ATGCTTGTCAAAGGTAATAAATCTGGAGAT
Sp2228F	ACGTTGAGGGGTTAGTTGCTGCTGGTCTGG
Sp154F	GATATTTGGAAAGATTCTGATTTTGGTAAG
Sp2312F	CTGTTATATCAACAATTACCAAGTCTGCTG
Sp2293F	TTTTCTACTTTATTTCCTACTTCATTTGTT
Sp328F	ATTACTTTCAAGATGTCTATGACTATATGC
Sp107F	ACCATCAAGGCTCTTATCTGCAGATTGTTA
Sp2325F	ATCGGCAATTTCTTGAAGACGTTCAGCGTT

Sp436F	CCATCCAACCATTTGATTTTGTGTAAATAT
Sp2211F	AAAATCTAAAAATACATTTTACCGTTTGAA
Sp2359F	AAGGATTTTACTTCAAAGATTTTTTTGTTG
Sp904bisF	GATATGGTGGACAGGTTCAGCAGGTCATGT
Sp2199F	TCAATATAAATCTGTCCAGTCTTTTTCTCA
Sp351F	ACAAACCTCTAATGGATAATATAGAACAAA
Sp348F	CGTTCTGCTTTAGTCATATGTGTCCTTTCT
Sp75F	AGAGTGTGTCCAAGACCAGAGTTACTGTTT
Sp379F	CATGCCTTTGAAAATAAATTCCGAGCCATT
Sp2132F	ATGGCTAATTATTGCGTGATGTTAGCGGTT
Sp299F	ATGGTCAGAAATCGAAGAAAACGAAGTCGT
Sp305bis	TTGGAAAAAACACGAAAGTGATATTACTTT
Sp263F	AAGTGAAGTTGAATTTTATTTGAGATACTA
Sp295F	TTGGAAAGATGGCAGAGGGGTTAACGCAGC
Sp2259F	TAATTTTGTTATATCTATATTTAAGTCTTT
Sp8R	TATCCATCTCGGTGAGATGAGAATTAGCTT
Sp31R	ATCAGTACCAACAAATGATTTTGTACCATC
Sp52R	TTTATTTTTTCTCAGTTCCTTGATTTTAGA
Sp36R	ACATATCCTTTTGTTAGGTCAAAAGAAGAT
Sp20R	AGATGTCTTGATATCAACTGCTTTAAAAGA
Sp830F	TCAAAAGTTTCCACTAATAGCGTTT
Sp298F	CTTTTAACCGTCTCTCCGCTTC
Sp39F	GCCTTTTCTAACTCTTCAG
Sp246F	GATTAGTTTGCGTACTCGCTC
Sp511F	GCTTGGGTTTGATAAGGG
Sp177R	AGTTACTGTTGAGGGTAGTCC
Sp87R	TATGCAAAGCAGTCACCAGC
Sp42R	TAAGTAACAAGACAGACTTGAA

Table S2. Terminal direct repeat sequences and distribution.

Sequence (5' → 3')		Number of isolates n (%)	Clonal complex or ST
GTTTTAGAGCTGTGCTGTTATTATGCTAGGACATCA	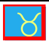	122 (59%)	CC1/CC19, CC10, ST103, ST243, ST1001
GTTTTAAAGCTGTGCTGTTATTATGCTAGGGCACCA	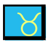	43 (21%)	CC23
GTTTTAGAGCTGTGCGGTTATTATGCTAGGGCACCG	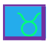	30 (15%)	CC17, ST130
GTTTTAGAGCTGCGCGGTTATTATGCTATGCTAGGA	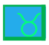	4 (2%)	CC22
GTTTTAGAGCTGTGCTGTTATTATGCTAGGGCACCA	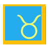	6 (3%)	ST26

Table S3. Altered leader sequences and distribution.

Leader end modification (n=)	CC/ST (n=)	Isolates
Last nucleotide deletion (15)	CC10 (13), CC19 (2)	142, 143, 211, 212, 361, 362, 431, 432, 439, 440, 488, 497, 498, 499, 500
Last nucleotide duplication (1)	CC10 (1)	508
26 additional nucleotides (1)	CC19 (1)	104
Single nucleotide difference (10)	ST130 (4), CC19 (2), CC22 (4)	24, 25, 43, 44, 116, 117, 121, 123, 420, 421

Figure S1. CRISPR1 spacer content of all (205) isolates from all (100) women. The CRISPR1 arrays are represented using a macro-enable Excel tool, whereby spacers are converted into two-color symbols based on spacer sequence. Gaps (= missing spacers) are shown with a boxed cross symbol ⊠ after alignment of identical spacers between strains of the same group. MLVA allelic profiles correspond to the number of repeats of each VNTR (SAG2, SAG3, SAG4, SAG7, SAG21, and SAG22). Terminal Direct Repeats (TDRs) are represented using a different color border according to their sequence (table S2). Isolates are grouped by source, and the period between sampling of isolates is indicated. Altered leader sequences are highlighted according to their differences (yellow: last nucleotide deletion, orange: last nucleotide duplication, red: 26 additional nucleotides, green: Single nucleotide difference). The spacer 247 (◆) is CC23 specific, is present in 82% of CC23 isolates and duplicate in 54% of them. **A**, isolates with “distinct” CRISPR1 arrays. **B**, isolates with “close” or “identical” CRISPR1 arrays.

Figure S1A

woman	strain	isolation date (mm/dd/yy)	* time between two isolates days (years)	MLVA Allelic profile	MLST	L
9	S.ag23	05/11/06	**1128 (3.1)**	3,3,3,5,0,2	ST19 (CC19)	gag
	S.ag22	06/11/09		3,3,1,0,7,4	ST23 (CC23)	gag
13	S.ag38	08/04/09	**987 (2.7)**	2,3,1,0,12,5	ST24 (CC23)	gag
	S.ag39	04/16/12		3,3,3,10,48,3	ST1 (CC1)	gag
16	S.ag45	04/28/11	**918 (2.5)**	3,3,3,6,19,3	ST136 (CC2)	gag
	S.ag46	10/31/13		3,3,2,0,9,6	ST26	gag
22	S.ag110	09/06/06	**1393 (3.8)**	3,3,2,6,11,1	ST1003	gag
	S.ag109	06/29/10		2,3,3,10,6,3	ST17 (CC17)	gag
27	S.ag136	08/25/06	**2373 (6.5)**	3,3,1,5,16,3	ST196 (CC2)	gag
	S.ag135	02/21/13		3,3,2,5,0,2	ST28 (CC19)	gag
29	S.ag141	11/15/07	**2056 (5.6)**	3,3,3,10,7,3	ST1 (CC1)	gag
	S.ag140	07/01/13		3,3,3,0,18,3	ST2 (CC2)	gag
32	S.ag153	09/14/07	**951 (2.6)**	3,3,1,10,11,3	ST1 (CC1)	gag
	S.ag152	04/21/10		4,3,3,0,11,5	ST23 (CC23)	gag
58	S.ag 329	10/06/05	**1568 (4.3)**	3,3,3,10,19,3	ST1 (CC1)	gag
	S.ag 328	01/20/10		4,3,1,10,19,5	ST23 (CC23)	gag
62	S.ag 348	04/29/08	**1019 (2.8)**	4,3,1,0,11,5	ST23 (CC23)	gag
	S.ag 346	02/11/11		3,3,2,6,0,2	ST327 (CC19)	gag
70	S.ag 396	04/10/08	**243 (0.7)**	3,3,3,5,0,2	ST19 (CC19)	gag
	S.ag 397	12/08/08		3,3,3,5,0,2	ST19 (CC19)	gag
73	S.ag 417	06/11/07	**1244 (3.4)**	3,3,3,10,7,3	ST1 (CC1)	gag
	S.ag 416	11/05/10		4,3,1,10,11,5	ST23 (CC23)	gag
89	S.ag 485	07/16/10	**906 (2.6)**	3,3,3,5,0,2	ST19 (CC19)	gag
	S.ag 487	01/06/13		3,3,3,10,27,3	ST1 (CC1)	gag
90	S.ag 490	07/05/07	**702 (1.9)**	3,3,3,6,2,7	ST27 (CC19)	gag
	S.ag 488	06/05/09		3,3,1,6,23,6	ST10 (CC10)	cga
94	S.ag 506	03/01/06	**2534 (6.9)**	3,3,1,6,5,7	ST12 (CC10)	gag
	S.ag 505	02/05/13		4,3,1,6,11,5	ST23 (CC23)	gag
95	S.ag 508	06/06/08	**1531 (4.3)**	3,3,1,6,0,7	ST12 (CC10)	agg
	S.ag 507	08/14/12		4,3,1,0,11,5	ST24 (CC23)	gag
96	S.ag 522	12/12/07	**1368 (3.7)**	3,3,3,10,40,3	ST1 (CC1)	gag
	S.ag 521	09/09/11		2,2,1,10,3,3	ST17 (CC17)	gag
98	S.ag 541	02/17/10	**371 (1.0)**	3,4,3,0,51,3	ST1005 (CC2)	gag
	S.ag 542	02/22/11		3,3,1,0,9,4	ST23 (CC23)	gag
100	S.ag 564	03/30/10	**1530 (4.2)**	3,4,4,10,18,3	ST297 (CC1)	gag
	S.ag 565	06/06/14		3,4,35,0,2	ST1001	gag

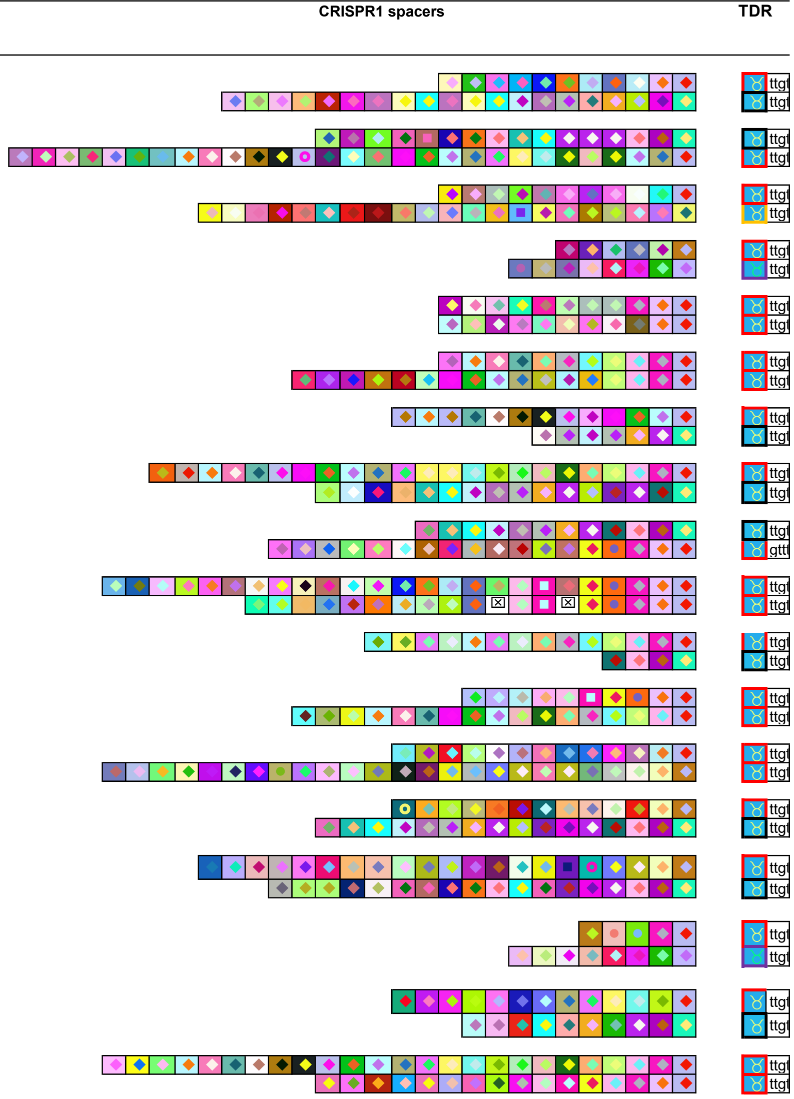

Figure S1B

woman	strain	isolation date (mm/dd/yy)	* time between two isolates days (years)	MLVA Allelic profile	MLST	L	TDR
1	S.ag1	01/21/10	908 (2.5)	3,3,3,5,0,2	ST19 (CC19)	gag	ttg
	S.ag2	07/17/12		3,3,3,5,0,2	ST19 (CC19)	gag	ttg
2	S.ag3	11/16/11	378 (1.0)	4,3,1,0,11,5	ST24 (CC23)	gag	ttg
	S.ag4	11/28/12		4,3,1,0,11,5	ST24 (CC23)	gag	ttg
3	S.ag8	02/26/07	135 (0.4)	3,3,1,0,10,5	ST23 (CC23)	gag	ttg
	S.ag9	07/11/07		3,3,1,0,10,5	ST23 (CC23)	gag	ttg
4	S.ag10	07/02/10	1376 (3.8)	3,3,3,5,0,2	ST182 (CC19)	gag	ttg
	S.ag12	04/08/14		3,3,3,5,0,2	ST182 (CC19)	gag	ttg
5	S.ag14	01/22/08	860 (2.4)	5,3,1,0,8,5	ST23 (CC23)	gag	ttg
	S.ag13	05/31/10		5,3,1,0,8,5	ST23 (CC23)	gag	ttg
6	S.ag15	11/16/09	750 (2.1)	3,3,1,0,10,2	ST23 (CC23)	gag	ttg
	S.ag16	12/06/11		3,3,1,0,10,2	ST23 (CC23)	gag	ttg
7	S.ag17	01/23/11	689 (1.9)	3,3,1,5,0,2	ST28 (CC19)	gag	ttg
	S.ag18	12/12/12		3,3,1,5,0,2	ST28 (CC19)	gag	ttg
8	S.ag20	09/26/13	172 (0.5)	3,3,3,5,2,7	ST27 (CC19)	gag	ttg
	S.ag21	03/17/14		3,3,3,5,2,7	ST27 (CC19)	gag	ttg
10	S.ag24	12/06/05	385 (1.1)	3,3,3,5,0,2	ST19 (CC19)	gag	ttg
	S.ag25	12/26/06		3,3,3,5,0,2	ST19 (CC19)	gag	ttg
11	S.ag28	12/22/09	923 (2.5)	3,3,3,5,0,2	ST19 (CC19)	gag	ttg
	S.ag30	07/02/12		3,3,3,5,0,2	ST19 (CC19)	gag	ttg
12	S.ag36	03/01/13	398 (1.1)	3,3,3,10,14,3	ST1 (CC1)	gag	ttg
	S.ag37	04/03/14		3,3,3,10,14,3	ST1 (CC1)	gag	ttg
14	S.ag40	05/05/09	1003 (2.7)	3,3,3,10,7,3	ST1 (CC1)	gag	ttg
	S.ag42	02/02/12		3,3,3,10,7,3	ST1 (CC1)	gag	ttg
15	S.ag44	04/04/06	1119 (3.1)	2,3,4,0,3,5	ST22 (CC22)	gag	cat
	S.ag43	04/27/09		2,3,4,0,3,5	ST22 (CC22)	gag	cat
17	S.ag88	05/23/07	201 (0.6)	3,3,1,5,0,2	ST28 (CC19)	gag	ttg
	S.ag90	12/10/07		3,3,1,5,0,2	ST28 (CC19)	gag	ttg
18	S.ag94	09/03/07	92 (0.3)	4,3,2,0,11,5	ST23 (CC23)	gag	ttg
	S.ag95	12/04/07		4,3,2,0,11,5	ST23 (CC23)	gag	ttg
19	S.ag96	11/22/09	787 (2.2)	3,3,1,5,0,2	ST28 (CC19)	ctct	ttg
	S.ag97	01/18/12		3,3,1,5,0,2	ST28 (CC19)	ctct	ttg
20	S.ag99	12/17/07	722 (2.0)	3,3,1,6,0,2	ST28 (CC19)	gag	ttg
	S.ag98	12/08/09		3,3,1,6,0,2	ST28 (CC19)	gag	ttg
21	S.ag106	03/21/06	1990 (5.5)	3,3,3,5,0,2	ST28 (CC19)	gag	ttg
	S.ag104	09/01/11		3,3,3,5,0,2	ST28 (CC19)	atg	ttg
23	S.ag111	09/25/09	96 (0.3)	3,3,1,0,8,4	ST366 (CC23)	gag	ttg
	S.ag113	12/30/09		3,3,1,0,8,4	ST366 (CC23)	gag	ttg
24	S.ag117	05/23/06	1871 (5.1)	3,3,1,15,9,4	ST130	gag	ttg
	S.ag116	07/07/11		3,3,1,15,9,4	ST130	gag	ttg
25	S.ag121	11/10/09	682 (1.9)	3,3,1,25,10,4	ST130	gag	ttg
	S.ag123	09/23/11		3,3,1,25,10,4	ST130	gag	ttg
26	S.ag128	01/05/11	322 (0.9)	2,2,2,10,6,3	ST17 (CC17)	gag	ttg
	S.ag132	11/23/11		2,2,2,10,6,3	ST17 (CC17)	gag	ttg
28	S.ag138	04/15/05	668 (1.8)	3,3,3,6,18,3	ST196 (CC2)	gag	ttg
	S.ag139	02/12/07	995 (2.7)	3,3,3,6,18,3	ST196 (CC2)	gag	ttg
	S.ag137	11/03/09		3,3,3,6,18,3	ST196 (CC2)	gag	ttg
30	S.ag142	02/18/13	667 (1.8)	3,3,1,6,20,6	ST10 (CC10)	cga	ttg
	S.ag143	12/17/14		3,3,1,6,20,6	ST10 (CC10)	cga	ttg
31	S.ag148	06/28/12	176 (0.5)	3,3,3,10,14,3	ST1 (CC1)	gag	ttg
	S.ag149	12/21/12		3,3,3,10,14,3	ST1 (CC1)	gag	ttg
33	S.ag161	02/15/08	2058 (5.6)	3,3,3,10,28,3	ST1 (CC1)	gag	ttg
	S.ag159	10/04/13		3,3,3,10,25,3	ST1 (CC1)	gag	ttg
34	S.ag169	01/19/06	1273 (3.5)	3,3,1,0,32,3	ST2 (CC2)	gag	ttg
	S.ag168	07/15/09		3,3,1,0,27,3	ST2 (CC2)	gag	ttg
35	S.ag171	09/11/06	940 (2.6)	3,3,1,0,14,5	ST1002 (CC23)	gag	ttg
	S.ag170	04/08/09		3,3,1,0,19,5	ST1002 (CC23)	gag	ttg
36	S.ag183	07/18/13	112 (0.3)	3,3,1,0,12,4	ST144 (CC23)	gag	ttg
	S.ag185	11/07/13		3,3,1,0,12,4	ST144 (CC23)	gag	ttg
37	S.ag195	08/08/05	122 (0.3)	3,3,3,6,18,3	ST3 (CC1)	gag	ttg
	S.ag196	12/08/05		3,3,3,6,18,3	ST3 (CC1)	gag	ttg
38	S.ag200	02/07/11	1092 (3.0)	3,3,3,5,0,2	ST19 (CC19)	gag	ttg
	S.ag202	02/03/14		3,3,3,5,0,2	ST19 (CC19)	gag	ttg
39	S.ag207	03/01/06	120 (0.3)	2,2,2,10,8,3	ST17 (CC17)	gag	ttg
	S.ag209	06/29/06		2,2,2,10,8,3	ST17 (CC17)	gag	ttg
40	S.ag211	05/10/07	98 (0.3)	2,2,2,10,8,3	ST12 (CC10)	cga	ttg
	S.ag212	08/16/07		3,3,1,6,0,3	ST12 (CC10)	cga	ttg
41	S.ag228	04/16/09	559 (1.5)	3,3,3,10,10,3	ST1 (CC1)	gag	ttg
	S.ag230	10/27/10		3,3,3,10,10,3	ST1 (CC1)	gag	ttg
42	S.ag231	08/20/09	637 (1.7)	4,3,1,0,11,5	ST23 (CC23)	gag	ttg
	S.ag232	05/19/11		4,3,1,0,11,5	ST23 (CC23)	gag	ttg
43	S.ag234	10/01/07	1117 (3.1)	2,2,2,10,8,3	ST17 (CC17)	gag	ttg
	S.ag233	10/22/10		2,2,2,10,8,3	ST17 (CC17)	gag	ttg
44	S.ag236	03/03/09		2,2,2,12,8,3	ST17 (CC17)	gag	ttg

CRISPR1 spacers

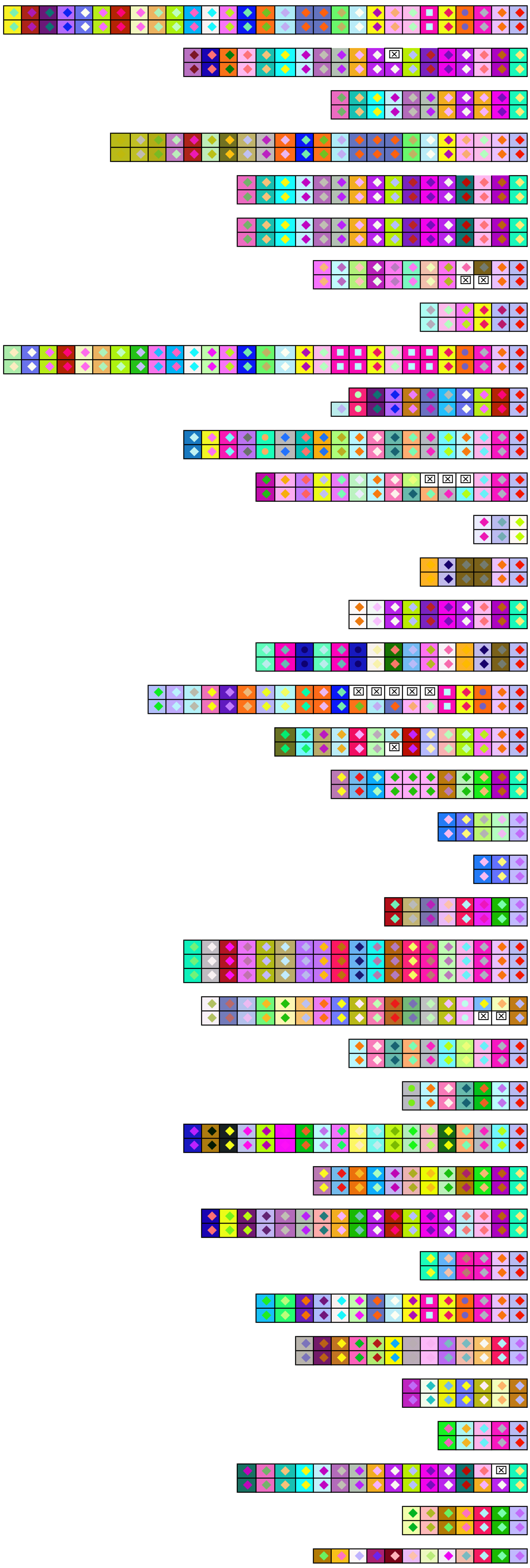

	S.ag235	12/03/12	1371 (3.8)	2,2,2,12,8,3	ST17 (CC17)	gag	ttg
45	S.ag247	06/01/10	155 (0.4)	3,3,1,6,14,5	ST12 (CC10)	gag	ttg
	S.ag248	11/03/10		3,3,1,6,14,5	ST12 (CC10)	gag	ttg
46	S.ag249	08/23/05	265 (0.7)	3,3,2,10,7,3	ST1 (CC1)	gag	ttg
	S.ag254	05/15/06		3,3,2,10,7,3	ST1 (CC1)	gag	ttg
47	S.ag259	03/27/08	1342 (3.7)	3,3,3,5,0,2	ST19 (CC19)	gag	ttg
	S.ag258	11/29/11		3,3,3,5,0,2	ST19 (CC19)	gag	ttg
48	S.ag274	08/20/12	129 (0.4)	3,3,2,0,9,7	ST26	gag	ttg
	S.ag275	12/27/12		3,3,2,0,9,7	ST26	gag	ttg
49	S.ag278	04/29/10	1140 (3.1)	3,3,3,5,0,2	ST19 (CC19)	gag	ttg
	S.ag279	06/12/13		3,3,3,5,0,2	ST19 (CC19)	gag	ttg
50	S.ag280	04/02/05	479 (1.3)	2,2,2,10,5,3	ST17 (CC17)	gag	ttg
	S.ag281	07/25/06		2,2,2,10,5,3	ST17 (CC17)	gag	ttg
51	S.ag287	03/23/07	1991 (5.5)	3,3,1,10,4,2	ST6 (CC10)	gag	ttg
	S.ag286	09/03/12		3,3,1,10,4,2	ST6 (CC10)	gag	ttg
52	S.ag289	02/04/08	1568 (4.3)	4,3,1,0,12,5	ST23 (CC23)	gag	ttg
	S.ag288	05/21/12		4,3,1,0,12,5	ST23 (CC23)	gag	ttg
53	S.ag293	05/03/13	483 (1.3)	4,3,1,0,11,5	ST23 (CC23)	gag	ttg
	S.ag295	08/29/14		4,3,1,0,11,5	ST23 (CC23)	gag	ttg
54	S.ag298	12/02/12	647 (1.8)	2,2,2,10,6,3	ST17 (CC17)	gag	ttg
	S.ag299	09/10/14		2,2,2,10,6,3	ST17 (CC17)	gag	ttg
55	S.ag303	07/17/12	132 (0.4)	3,2,3,10,3,3,	ST243	gag	ttg
	S.ag305	11/26/12		3,2,3,10,3,3,	ST243	cag	ttg
56	S.ag311	07/17/09	1055 (2.9)	3,3,2,0,11,5	ST23 (CC23)	gag	ttg
	S.ag313	06/06/12		3,3,2,0,11,5	ST23 (CC23)	gag	ttg
57	S.ag 325	04/24/05	1654 (4.5)	2,2,2,10,6,2	ST17 (CC17)	gag	ttg
	S.ag 324	11/03/09		2,2,2,10,6,2	ST17 (CC17)	gag	ttg
59	S.ag 330	05/19/11	540 (1.5)	3,3,3,6,16,10	ST196 (CC2)	gag	ttg
	S.ag 331	11/09/12		3,3,3,6,16,10	ST196 (CC2)	gag	ttg
60	S.ag 338	02/16/07	1326 (3.6)	2,2,2,10,5,3	ST17 (CC17)	gag	ttg
	S.ag 337	10/04/10		2,2,2,10,5,3	ST17 (CC17)	gag	ttg
61	S.ag 340	02/15/05	246 (0.7)	3,3,3,4,0,2	ST19 (CC19)	gag	ttg
	S.ag 343	10/19/05		3,3,3,4,0,2	ST19 (CC19)	gag	ttg
63	S.ag 358	07/30/08	705 (1.9)	3,3,1,5,9,2	ST6 (CC10)	gag	ttg
	S.ag 357	07/05/10		3,3,1,5,9,2	ST6 (CC10)	gag	ttg
64	S.ag 362	08/19/08	268 (0.7)	3,3,1,5,0,2	ST28 (CC19)	cga	ttg
	S.ag 361	05/14/09		3,3,1,5,0,2	ST28 (CC19)	cga	ttg
65	S.ag 365	10/12/09	935 (2.6)	3,3,3,10,23,3	ST1 (CC1)	gag	ttg
	S.ag 366	05/04/12		3,3,3,10,23,3	ST1 (CC1)	gag	ttg
66	S.ag 371	01/08/09	134 (0.4)	3,3,3,6,15,3	ST196 (CC2)	gag	ttg
	S.ag 372	05/22/09		3,3,3,6,15,3	ST196 (CC2)	gag	ttg
67	S.ag 380	02/03/04		4,3,1,0,8,5	ST23 (CC23)	gag	ttg
	S.ag 381	11/12/04	283 (0.8)	4,3,1,0,8,5	ST23 (CC23)	gag	ttg
	S.ag 382	01/07/06	421 (1.2)	4,3,1,0,8,5	ST23 (CC23)	gag	ttg
68	S.ag 387	09/29/11	406 (1.1)	4,3,2,0,11,5	ST23 (CC23)	gag	ttg
	S.ag 388	11/08/12		4,3,2,0,11,5	ST23 (CC23)	gag	ttg
69	S.ag 391	06/29/09	739 (2.0)	3,3,3,10,22,3	ST1 (CC1)	gag	ttg
	S.ag 393	07/08/11		3,3,3,10,22,3	ST1 (CC1)	gag	ttg
71	S.ag 400	12/08/10	434 (1.2)	3,3,3,6,0,2	ST19 (CC19)	gag	ttg
	S.ag 401	02/15/12		3,3,3,6,0,2	ST19 (CC19)	gag	ttg
72	S.ag 409	02/01/08	768 (2.1)	3,3,3,10,19,3	ST1 (CC1)	gag	ttg
	S.ag 408	03/10/10		3,3,3,10,19,3	ST1 (CC1)	gag	ttg
74	S.ag 421	03/20/08	1274 (3.5)	2,3,6,0,4,5	ST22 (CC22)	gag	cat
	S.ag 420	09/15/11		2,3,6,0,4,5	ST22 (CC22)	gag	cat
75	S.ag 427	07/28/05	935 (2.6)	3,3,3,10,7,3	ST1 (CC1)	gag	ttg
	S.ag 428	02/18/08		3,3,3,10,52,3	ST1 (CC1)	gag	ttg
76	S.ag431	01/26/10	694 (1.9)	3,3,1,6,14,7	ST7 (CC10)	cga	ttg
	S.ag432	12/21/11		3,3,1,6,14,7	ST7 (CC10)	cga	ttg
77	S.ag433	07/24/10	183 (0.5)	3,3,1,0,12,4	ST23 (CC23)	gag	ttg
	S.ag434	01/23/11		3,3,1,0,12,4	ST23 (CC23)	gag	ttg
78	S.ag 435	07/30/09	848 (2.3)	3,3,3,6,0,2	ST19 (CC19)	gag	ttg
	S.ag 436	11/25/11		3,3,3,6,0,2	ST19 (CC19)	gag	ttg
79	S.ag 439	01/10/10	960 (2.6)	3,3,1,7,14,3	ST8 (CC10)	cga	ttg
	S.ag 440	08/27/12		3,3,1,7,14,3	ST8 (CC10)	cga	ttg
80	S.ag 442	10/02/06	639 (1.8)	3,3,3,10,7,3	ST1 (CC1)	gag	ttg
	S.ag 443	07/02/08		3,3,3,10,7,3	ST1 (CC1)	gag	ttg
81	S.ag 444	11/02/07		3,3,2,6,0,2	ST327 (CC19)	gag	ttg
	S.ag 446	09/22/08	325 (0.9)	3,3,2,6,0,2	ST327 (CC19)	gag	ttg
	S.ag 449	09/11/09	354 (1.0)	3,3,2,6,0,2	ST327 (CC19)	gag	ttg
82	S.ag 456	12/09/08	224 (0.6)	2,3,1,0,26,5	ST88 (CC23)	gag	ttg
	S.ag 454	07/21/09		2,3,1,0,26,5	ST88 (CC23)	gag	ttg
83	S.ag 459	02/19/08	1224 (3.4)	3,3,3,10,7,3	ST1 (CC1)	gag	ttg
	S.ag 457	06/27/11		3,3,3,10,7,3	ST1 (CC1)	gag	ttg
84	S.ag 463	12/17/08	616 (1.7)	2,2,2,10,6,3	ST17 (CC17)	gag	ttg
	S.ag 462	08/25/10		2,2,2,10,6,3	ST17 (CC17)	gag	ttg
85	S.ag 469	08/30/05	1862 (5.1)	3,3,1,0,12,4	ST23 (CC23)	gag	ttg
	S.ag 468	10/05/10		3,3,1,0,12,4	ST23 (CC23)	gag	ttg
86	S.ag 471	08/12/05		3,3,3,10,8,3	ST1 (CC1)	gag	ttg

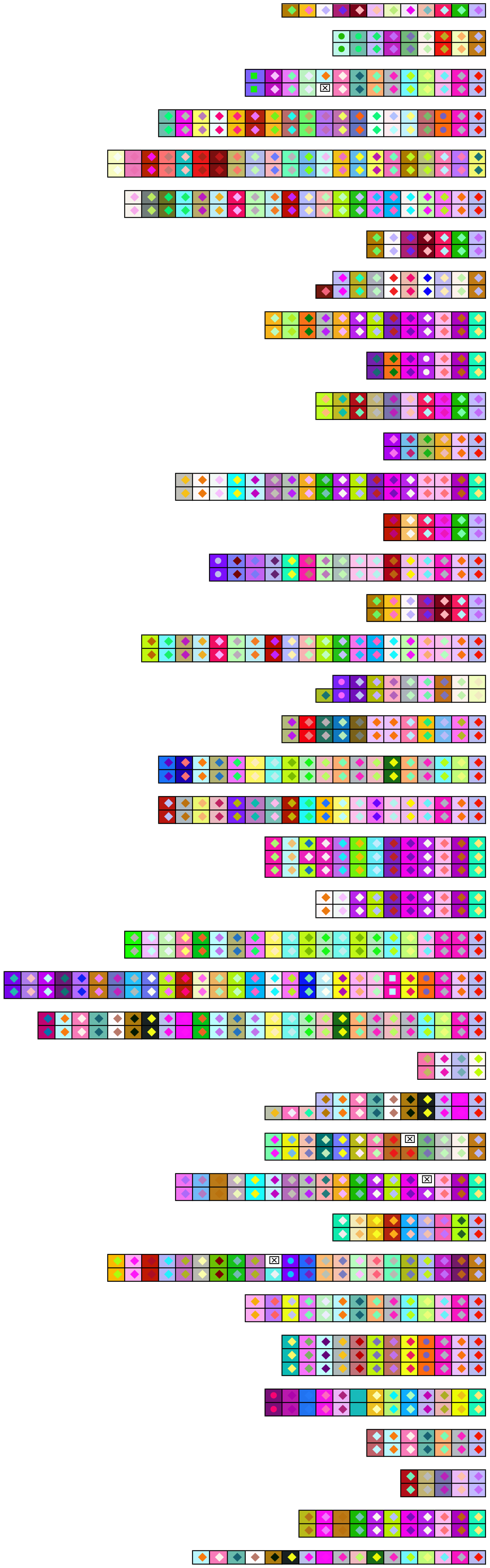

	S.ag 472	12/11/07	**851 (2.3)**	3,3,3,10,8,3	ST1 (CC1)	gag	ttgt
87	S.ag 473	11/16/09		2,2,2,10,6,3	ST17 (CC17)	gag	ttgt
	S.ag 475	05/20/10	**185 (0.5)**	2,2,2,10,6,3	ST17 (CC17)	gag	ttgt
88	S.ag 477	10/05/05		3,3,3,5,0,2	ST19 (CC19)	gag	ttgt
	S.ag 476	12/25/09	**1542 (4.2)**	3,3,3,5,0,2	ST19 (CC19)	gag	ttgt
91	S.ag 492	06/26/08		2,2,1,10,6,3	ST1004 (CC17)	gag	ttgt
	S.ag 491	10/10/12	**1567 (4.3)**	2,2,1,10,6,3	ST1004 (CC17)	gag	ttgt
92	S.ag 498	10/09/08		3,3,1,6,13,3	ST8 (CC10)	cga	ttgt
	S.ag 497	07/06/10	**635 (1.7)**	3,3,1,6,13,3	ST8 (CC10)	cga	ttgt
93	S.ag 499	05/12/05		3,3,1,6,9,7	ST10 (CC10)	cga	ttgt
	S.ag 500	05/31/06	**384 (1.1)**	3,3,1,6,9,7	ST10 (CC10)	cga	ttgt
97	S.ag 536	05/18/09		3,5,2,0,27,7	ST26	gag	ttgt
	S.ag 537	06/01/12	**1110 (3.0)**	3,5,2,0,27,7	ST26	gag	ttgt
	S.ag 538	02/12/13	**256 (0.7)**	3,5,2,0,27,7	ST26	gag	ttgt
98	S.ag 544	11/08/07		3,4,3,0,39,3	ST1005 (CC2)	gag	ttgt
	S.ag 541	02/17/10	**832 (2.3)**	3,4,3,0,51,3	ST1005 (CC2)	gag	ttgt
99	S.ag 545	05/22/12		2,3,2,10,6,3	ST17 (CC17)	gag	ttgt
	S.ag 546	02/10/14	**629 (1.7)**	2,3,2,10,6,3	ST17 (CC17)	gag	ttgt

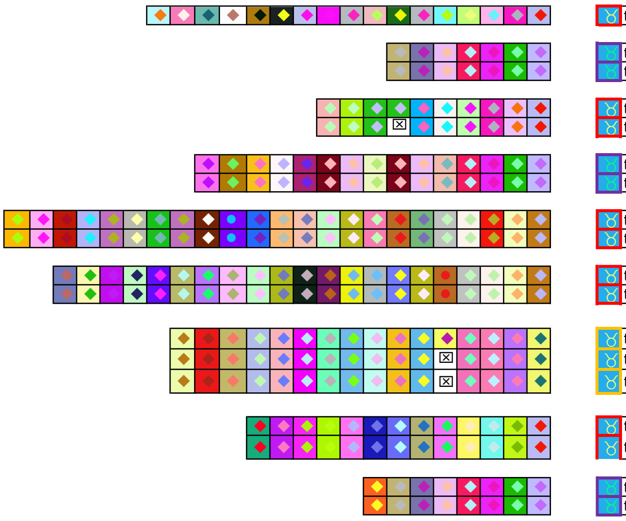

Figure S2. DNA sequences of CRISPR1 arrays represented in Figure 3. Leader and spacer sequences are represented by lowercase letters, while Direct Repeats (DRs) are represented by uppercase letters.

Woman 2

Isolate 3

AaactgaagtcctgctgagacgaatggcgcgattacgaaagctcaaaagaaaattttctacgagGTTTTAGAGCTGTGCTGTT
TCGAATGGTTCCAAAACaactacagagattgtctttagagccgttaaGTTTTAGAGCTGTGCTGTTTCGAATGGTTCCAAAAC
gatatggtggacaggttcagcaggtcatgtGTTTTAGAGCTGTGCTGTTTCGAATGGTTCCAAAACtatcgcaaatgaaacgg
gcgcttttgcactGTTTTAGAGCTGTGCTGTTTCGAATGGTTCCAAAACttaataactgtccgttttgccatttcttgcGTTT
TAGAGCTGTGCTGTTTCGAATGGTTCCAAAACgaagtatactacggttagagattggctcaaGTTTTAGAGCTGTGCTGTTTC
GAATGGTTCCAAAACgaaacttcgattagtttgcgtactcgctcaGTTTTAGAGCTGTGCTGTTTCGAATGGTTCCAAAACat
tagtcctgttgttatgatggatgattatGTTTTAGAGCTGTGCTGTTTCGAATGGTTCCAAAACacaaacctctaatggataa
tatagaacaaaGTTTTAGAGCTGTGCTGTTTCGAATGGTTCCAAAACacacaattaaacataaagaagtgcttatttGTTTTA
GAGCTGTGCTGTTTCGAATGGTTCCAAAACtcaggagattgtgtgtactcacgaattttttGTTTTAGAGCTGTGCTGTTTCGA
ATGGTTCCAAAACaaattgtttttgttgtaatataaagtcgacGTTTTAGAGCTGTGCTGTTTCGAATGGTTCCAAAACaacc
ttgataggcgtactgtatggattccaGTTTTAGAGCTGTGCTGTTTCGAATGGTTCCAAAACctactgtattatctgaaatag
tatcgttttGTTTTAGAGCTGTGCTGTTTCGAATGGTTCCAAAACtctcgggaaatgcaactgatgctgcaatcaGTTTTAGA
GCTGTGCTGTTTCGAATGGTTCCAAAACaaattgtttttgttgtaatataaagtcgacGTTTTAGAGCTGTGCTGTTTCGAAT
GGTTCCAAAACttaataactgtccgttttgccatttcttgcGTTTTAGAGCTGTGCTGTTTCGAATGGTTCCAAAACagaggg
gaaaatatcaatgccgaatgctgaGTTTTAGAGCTGTGCTGTTTCGAATGGTTCCAAAACgatggtacaaaatcatttgttgg
tactgatGTTTTAAAGCTGTGCTGTTATTATGCTAGGGCACCAttgtggtgttctagtttttttgttatactgaaataaatttt
cagagaatgtgggggaaggcggtaattagattaattcaagacgtaattcagaacttagttggccaagctaacgaaatcacccc
aatttatcagtttgattgggaaacttatatattggcgactaaaaaatatgaacgtcatttagaggtgtgtctattagtagaaa
attcgaattgtttttcggattcaaagagaatgtgtcgataaaagagatatgaaaggctataatt

Isolate 4

Acaattctgataggcgaaatatggtatactattagtaaaagcacagtaataacaaggaatcatcgaaactgaagtcctgctga
gacgaatggcgcgattacgaaagctcaaaagaaaattttctacgagGTTTTAGAGCTGTGCTGTTTCGAATGGTTCCAAAACa
actacagagattgtctttagagccgttaaGTTTTAGAGCTGTGCTGTTTCGAATGGTTCCAAAACgatatggtggacaggttc
agcaggtcatgtGTTTTAGAGCTGTGCTGTTTCGAATGGTTCCAAAACtatcgcaaatgaaacgggcgcttttgcactGTTTT
AGAGCTGTGCTGTTTCGAATGGTTCCAAAACttaataactgtccgttttgccatttcttgcGTTTTAGAGCTGTGCTGTTTCG
AATGGTTCCAAAACgaagtatactacggttagagattggctcaaGTTTTAGAGCTGTGCTGTTTCGAATGGTTCCAAAACgaa
acttcgattagtttgcgtactcgctcaGTTTTAGAGCTGTGCTGTTTCGAATGGTTCCAAAACattagtcctgttgttatgat
ggatgattatGTTTTAGAGCTGTGCTGTTTCGAATGGTTCCAAAACacaaacctctaatggataatatagaacaaaGTTTTAG
AGCTGTGCTGTTTCGAATGGTTCCAAAACacacaattaaacataaagaagtgcttatttGTTTTAGAGCTGTGCTGTTTCGAA
TGGTTCCAAAACtcaggagattgtgtgtactcacgaattttttGTTTTAGAGCTGTGCTGTTTCGAATGGTTCCAAAACaaatt
gtttttgttgtaatataaagtcgacGTTTTAGAGCTGTGCTGTTTCGAATGGTTCCAAAACaaattgtttttgttgtaatata
aagtcgacGTTTTAGAGCTGTGCTGTTTCGAATGGTTCCAAAACaaccttgataggcgtactgtatggattccaGTTTTAGAG
CTGTGCTGTTTCGAATGGTTCCAAAACCtactgtattatctgaaatagtatcgttttGTTTTAGAGCTGTGCTGTTTCGAATG
GTTCCAAAACtctcgggaaatgcaactgatgctgcaatcaGTTTTAGAGCTGTGCTGTTTCGAATGGTTCCAAAACaaattgt
ttttgttgtaatataaagtcgacGTTTTAGAGCTGTGCTGTTTCGAATGGTTCCAAAACttaataactgtccgttttgccatt
tcttgcGTTTTAGAGCTGTGCTGTTTCGAATGGTTCCAAAACagaggggaaaatatcaatgccgaatgctgaGTTTTAGAGCT
GTGCTGTTTCGAATGGTTCCAAAACgatggtacaaaatcatttgttggtactgatGTTTTAAAGCTGTGCTGTTATTATGCTA
GGGCACCAttgtggtgttctagtttttttgttatactgaaataaattttcagagaatgtgggggaaggcggtaattagattaat
tcaagacgtaattcagaacttagttggccaagctaacgaaatcaccccaatttatcagtttgattgggaaacttatatattgg
cgactaaaaaatatgaacgtcatttagaggtgtgtctattagtagaaaattcgaattgtttttcggattcaaagagaatgtgt
cgataaaagagatatgaaaggctataattccaaccttatggttaaagggcaagatgtc

Woman 7

Isolate 17

tttcttgataggcgatatatggtataatattagtaaaagcacagtaataacaaggaatcatcgaaactgaagtcctgctgaga
cgaatggcgcgattacgaaagctcaaaagaaaattttctacgagGTTTTAGAGCTGTGCTGTTTCGAATGGTTCCAAAACtac
gtcattatttgttactaaaggactcatGTTTTAGAGCTGTGCTGTTTCGAATGGTTCCAAAACattggttattttacaaacga
cccggataaaGTTTTAGAGCTGTGCTGTTTCGAATGGTTCCAAAACacaaatccagcttacttttaatcaaaagctGTTTTAG
AGCTGTGCTGTTTCGAATGGTTCCAAAACaaaacgtcgtaaacgtgtcattgattgtatGTTTTAGAGCTGTGCTGTTTCGAA
TGGTTCCAAAACttttgctaagtctgcaacgcctttaataatGTTTTAGAGCTGTGCTGTTTCGAATGGTTCCAAAACctttg
taaaaatttctttaactaattcatgGTTTTAGAGCTGTGCTGTTTCGAATGGTTCCAAAACtctcgatggaagatttcgaatt
aaaaaccaGTTTTAGAGCTGTGCTGTTTCGAATGGTTCCAAAACtttaacaaaatcataacctaaaggggtattGTTTTAGAG
CTGTGCTGTTTCGAATGGTTCCAAAACtattatcagatacgctaaaaccagtagttcGTTTTAGAGCTGTGCTGTTTCGAATG
GTTCCAAAACctacacgcaagattacatggcttctcagttGTTTTAGAGCTGTGCTGTTTCGAATGGTTCCAAAACtggttat
acatttactaatccatcagcattGTTTTAGAGCTGTGCTGTTTCGAATGGTTCCAAAACaagctaattctcatctcaccgaga
tggataGTTTTAGAGCTGTGCTGTTATTATGCTAGGACATCAttgtggtgttctagtttttttgttatactgaaataaatttc
agagaatgtgggggaaggcggtaattagattaattcaagacgtaattcagaacttagttggccaagctaacgaaatcacccca
atttatcagtttgattgggaaacttatatattggcgactaaaaaatatgaacgtcatttagaggtgtgtctattagtagaaaa

ttcgaattgtttttcggattcaaagagaatgtgtcaataaaagagatatgaaaggctataattccaaccttatggttaaaggg
ctaggtgtctacgc

Isolate 18

tttcttgataggcgaaatatggtataatattagtaaaagcacagtaataacaaggaatcatcgaaactgaagtcctgctgaga
cgaatggcgcgattacgaaagctcaaaagaaaatttctacgagGTTTTAGAGCTGTGCTGTTTCGAATGGTTCCAAAACtac
gtcattatttgttactaaaggactcatGTTTTAGAGCTGTGCTGTTTCGAATGGTTCCAAAACattggttattttacaaacga
cccggataaaGTTTTAGAGCTGTGCTGTTTCGAATGGTTCCAAAACacaaatccagcttacttttaatcaaaagctGTTTTAG
AGCTGTGCTGTTTCGAATGGTTCCAAAACaaaacgtcgtaaacgtgtcattgattgtatGTTTTAGAGCTGTGCTGTTTCGAA
TGGTTCCAAAACttttgctaagtctgcaacgcctttaataatGTTTTAGAGCTGTGCTGTTTCGAATGGTTCCAAAACctttg
taaaaatttctttaactaattcatgGTTTTAGAGCTGTGCTGTTTCGAATGGTTCCAAAACtctcgatggaagatttcgaatt
aaaaaccaGTTTTAGAGCTGTGCTGTTTCGAATGGTTCCAAAACtttaacaaaatcataacctaaaggggtattGTTTTAGAG
CTGTGCTGTTTCGAATGGTTCCAAAACtggttatacatttactaatccatcagcattGTTTTAGAGCTGTGCTGTTTCGAATG
GTTCCAAAACaagctaattctcatctcaccgagatggataGTTTTAGAGCTGTGCTGTTATTATGCTAGGACATCAttgtggt
gttctagtttttgttatactgaaataaattttcagagaatgtgggggaaggcggtaattagattaattcaagacgtaattca
gaacttagttggccaagctaacgaaatcaccccaatttatcagtttgattgggaaacttatatattggcgactaaaaaatatg
aacgtcatttagaggtgtgtctattagtagaaaattcgaattgtttttcggattcaaagagaatgtgtcaataaaagagatat
gaaaggctataattccaaccttatggttaaagggctaggtgtctacgc

Woman 11

Isolate 28

atatggtataatattagtaaaagcacagtaataacaaggaatcatcgaaactgaagtcctgctgagacgaatggcgcgattac
gaaagctcaaaagaaaatttctacgagGTTTTAGAGCTGTGCTGTTTCGAATGGTTCCAAAACtactggtcttctgaaaaag
ttttataactGTTTTAGAGCTGTGCTGTTTCGAATGGTTCCAAAACcctccgacttctggcgcagtcattgcaactGTTTTAG
AGCTGTGCTGTTTCGAATGGTTCCAAAACacatgccaccttactgcatggtatatcgttGTTTTAGAGCTGTGCTGTTTCGAA
TGGTTCCAAAACataatcttctgaaattgttgcttcgtaataGTTTTAGAGCTGTGCTGTTTCGAATGGTTCCAAAACccatc
caaccatttgattttgtgtaaatatGTTTTAGAGCTGTGCTGTTTCGAATGGTTCCAAAACgttgcatgaattgaaagttcaa
tataagcaGTTTTAGAGCTGTGCTGTTTCGAATGGTTCCAAAACccgttcaatctgttcttgcttttggtcatcGTTTTAGAG
CTGTGCTGTTTCGAATGGTTCCAAAACaagggtgttagatgataataccttttttaaGTTTTAGAGCTGTGCTGTTTCGAATG
GTTCCAAAACacaccgttgcggttgttgtcggtcactcaaGTTTTAGAGCTGTGCTGTTTCGAATGGTTCCAAAACaagctaa
ttctcatctcaccgagatggataGTTTTAGAGCTGTGCTGTTATTATGCTAGGACATCAttgtggtgttctagtttttgtta
tactgaaataaattttcagagaatgtgggggaaggcggtaattagattaattcaagacgtaattcagaacttagttggccaag
ctaacgaaatcaccccaatttatcagtttgattgggaaacttatatattggcgactaaaaaatatgaacgtcatttagaggtg
tgtctattagtagaaaattcgaattgtttttcggattcaaagagaatgtgtcaataaaagagatatgaaaggctataattcca
accttatggtaaagggctaggatgtcta

Isolate 30

tatggtataatattagtaaaagcacagtaataacaaggaatcatcgaaactgaagtcctgctgagacgaatggcgcgattacg
aaagctcaaaagaaaatttctacgagGTTTTAGAGCTGTGCTGTTTCGAATGGTTCCAAAACaaaaatgaagtgcgaaaaaa
actactcgatGTTTTAGAGCTGTGCTGTTTCGAATGGTTCCAAAACtactggtcttctgaaaaagttttataactGTTTTAGA
GCTGTGCTGTTTCGAATGGTTCCAAAACcctccgacttctggcgcagtcattgcaactGTTTTAGAGCTGTGCTGTTTCGAAT
GGTTCCAAAACacatgccaccttactgcatggtatatcgttGTTTTAGAGCTGTGCTGTTTCGAATGGTTCCAAAACataatc
ttctgaaattgttgcttcgtaataGTTTTAGAGCTGTGCTGTTTCGAATGGTTCCAAAACccatccaaccatttgattttgtg
taaatatGTTTTAGAGCTGTGCTGTTTCGAATGGTTCCAAAACgttgcatgaattgaaagttcaatataagcaGTTTTAGAGC
TGTGCTGTTTCGAATGGTTCCAAAACccgttcaatctgttcttgcttttggtcatcGTTTTAGAGCTGTGCTGTTTCGAATGG
TTCCAAAACaagggtgttagatgataataccttttttaaGTTTTAGAGCTGTGCTGTTTCGAATGGTTCCAAAACacaccgtt
gcggttgttgtcggtcactcaaGTTTTAGAGCTGTGCTGTTTCGAATGGTTCCAAAACaagctaattctcatctcaccgagat
ggataGTTTTAGAGCTGTGCTGTTATTATGCTAGGACATCAttgtggtgttctagtttttgttatactgaaataaattttca
gagaatgtgggggaaggcggtaattagattaattcaagacgtaattcagaacttagttggccaagctaacgaaatcaccccaa
tttatcagtttgattgggaaacttatatattggcgactaaaaaatatgaacgtcatttagaggtgtgtctattagtagaaaat
tcgaattgtttttcggattcaaagagaatgtgtcaataaaagagatatgaaaggctataattccaaccttatggtaaagggct
aggtgtctacg

Woman 14

Isolate 40

tatggtataatattagtaaaagcacagtaataacaaggaatcatcgaaactgaagtcctgctgagacgaatggcgcgattacg
aaagctcaaaagaaaatttctacgagGTTTTAGAGCTGTGCTGTTTCGAATGGTTCCAAAACattaagccaaaggcggttaa
aacaggaatgGTTTTAGAGCTGTGCTGTTTCGAATGGTTCCAAAACttgataggcttcagttaccaagatagcacgGTTTTAG
AGCTGTGCTGTTTCGAATGGTTCCAAAACatgttctgagttacctttctcgacacgcggGTTTTAGAGCTGTGCTGTTTCGAA
TGGTTCCAAAACtgtttttattgaaatatccaataggtctgaGTTTTAGAGCTGTGCTGTTTCGAATGGTTCCAAAACtggaa
ctgaatatcactcgcttttacatcGTTTTAGAGCTGTGCTGTTTCGAATGGTTCCAAAACaatcgtcttcattgttgcgatg
attttgatGTTTTAGAGCTGTGCTGTTTCGAATGGTTCCAAAACaacattagccttttctaactcttcagctgtGTTTTAGAG
CTGTGCTGTTTCGAATGGTTCCAAAACtatgtcttctaacagttgcttcttgtgcttGTTTTAGAGCTGTGCTGTTTCGAATG
GTTCCAAAACatatgttccactctatgaatttaggctcatGTTTTAGAGCTGTGCTGTTTCGAATGGTTCCAAAACtttttac
caatgcttccatatcgcttatatGTTTTAGAGCTGTGCTGTTTCGAATGGTTCCAAAACtacttgacgaattgaagatgacgg
aatttaGTTTTAGAGCTGTGCTGTTTCGAATGGTTCCAAAACaagctaattctcatctcaccgagatggataGTTTTAGAGCT
GTGCTGTTATTATGCTAGGACATCAttgtggtgttctagtttttgttatactgaaataaattttcagagaatgtgggggaag

gcggtaattagattaattcaagacgtaattcagaacttagttggccaagctaacgaaatcaccccaatttatcagtttgattg
ggaaacttatatattggcgactaaaaaatatgaacgtcatttagaggtgtgtctattagtagaaaattcgaattgtttttcgg
attcaaagagaatgtgtcaataaaagagatatgaaaggctataattccaaccttatggt

Isolate 42
gaatatggtataatattagtaaaagcacagtaataacaaggaatcatcgaaactgaagtcctgctgagacgaatggcgcgatt
acgaaagctcaaaagaaaattttctacgagGTTTTAGAGCTGTGCTGTTTCGAATGGTTCCAAAACattaagccaaaggcggt
taaaacaggaatgGTTTTAGAGCTGTGCTGTTTCGAATGGTTCCAAAACttgataggcttcagttaccaagatagcacgGTTT
TAGAGCTGTGCTGTTTCGAATGGTTCCAAAACatgttctgagttacctttctcgacacgcggGTTTTAGAGCTGTGCTGTTTC
GAATGGTTCCAAAACtgtttttattgaaatatccaataggtctgaGTTTTAGAGCTGTGCTGTTTCGAATGGTTCCAAAACtg
gaactgaatatcactcgctttttacatcGTTTTAGAGCTGTGCTGTTTCGAATGGTTCCAAAACaatcgtcttcattgttgcg
atgattttgatGTTTTAGAGCTGTGCTGTTTCGAATGGTTCCAAAACaacattagcctttttctaactcttcagctgtGTTTTA
GAGCTGTGCTGTTTCGAATGGTTCCAAAACtatgtcttctaacagttgcttcttgtgcttGTTTTAGAGCTGTGCTGTTTCGA
ATGGTTCCAAAACccgtcaaacaagagcgacagcgaaacaagcGTTTTAGAGCTGTGCTGTTTCGAATGGTTCCAAAACTtaa
cagtttcaagtctgtcttgttacttaGTTTTAGAGCTGTGCTGTTTCGAATGGTTCCAAAACactctaaatgatagttatgag
ttaaatgttGTTTTAGAGCTGTGCTGTTTCGAATGGTTCCAAAACcaaattacagtttcgactgattatggaaatGTTTTAGA
GCTGTGCTGTTTCGAATGGTTCCAAAACtttttaccaatgcttccatatcgcttatatGTTTTAGAGCTGTGCTGTTTCGAAT
GGTTCCAAAACtacttgacgaattgaagatgacggaatttaGTTTTAGAGCTGTGCTGTTTCGAATGGTTCCAAAACaagcta
attctcatctcaccgagatggataGTTTTAGAGCTGTGCTGTTATTATGCTAGGACATCAttgtggtgttctagtttttttgtt
atactgaaataaattttcagagaatgtgggggaaggcggtaattagattaattcaagacgtaattcagaacttagttggccaa
gctaacgaaatcaccccaatttatcagtttgattgggaaacttatatattggcgactaaaaaatatgaacgtcatttagaggt
gtgtctattagtagaaaattcgaattgtttttcggattcaaagagaatgtgtcaataaaagagatatgaaaggctataattcc
aaccttatggtaaagggctaggtgt

Woman 20

Isolate 98
cgaaatatggtataatattagtaaaagcacagtaataacaaggaatcatcgaaactgaagtcctgctgagacgaatggcgcga
ttacgaaagctcaaaagaaaattttctacgagGTTTTAGAGCTGTGCTGTTTCGAATGGTTCCAAAACacaaaatcttttgtt
gctcctggacgtattGTTTTAGAGCTGTGCTGTTTCGAATGGTTCCAAAACatgtaaggtattgtaaaacttcttcttgcgGT
TTTAGAGCTGTGCTGTTTCGAATGGTTCCAAAACactgttcctataattaaaataaaagaggtaGTTTTAGAGCTGTGCTGTT
TCGAATGGTTCCAAAACtgttccagtaaaaagtaattttaaagcattGTTTTAGAGCTGTGCTGTTTCGAATGGTTCCAAAAC
cgctcgattgatgctatcaactatattgaaGTTTTAGAGCTGTGCTGTTTCGAATGGTTCCAAAACttcttcaagagaacttg
tagaacagcttcaGTTTTAGAGCTGTGCTGTTTCGAATGGTTCCAAAACaaggtacttttagcttgttcttgtggtgttGTTT
TAGAGCTGTGCTGTTTCGAATGGTTCCAAAACacagctactgtAAATTCTGCTTTTACGGTTGTTTTAGAGCTGTGCTGTTTC
GAATGGTTCCAAAACtagtgcagttgtcaaggagattgtgagcgaGTTTTAGAGCTGTGCTGTTTCGAATGGTTCCAAAACtt
taacctttgaaaatgtgaaaggctcgtaGTTTTAGAGCTGTGCTGTTTCGAATGGTTCCAAAACgcgatgatggtaagtcatc
atggacagcgtGTTTTAGAGCTGTGCTGTTTCGAATGGTTCCAAAACttttacacacgatgtcagatataatgtcaaGTTTTA
GAGCTGTGCTGTTTCGAATGGTTCCAAAACagtactgcactaggaattgtagagatcaaaGTTTTAGAGCTGTGCTGTTTCGA
ATGGTTCCAAAACcgtaccatctatcaatttaccgcaagctgtGTTTTAGAGCTGTGCTGTTTCGAATGGTTCCAAAACttaa
aagatttaaactatcaagcgtcaattGTTTTAGAGCTGTGCTGTTTCGAATGGTTCCAAAACTtctaaatgctggtgactgct
ttgcataaaGTTTTAGAGCTGTGCTGTTTCGAATGGTTCCAAAACTtgctgctagacccaaacagtttatttttagGTTTTAG
AGCTGTGCTGTTTCGAATGGTTCCAAAACtcctttttttagataatgtgcgatcacggacGTTTTAGAGCTGTGCTGTTTCGAA
TGGTTCCAAAACttttaccaatgcttccatatcgcttatatGTTTTAGAGCTGTGCTGTTTCGAATGGTTCCAAAACtggtta
tacatttactaatccatcagcattGTTTTAGAGCTGTGCTGTTTCGAATGGTTCCAAAACaagctaattctcatctcaccgag
atggataGTTTTAGAGCTGTGCTGTTATTATGCTAGGACATCAttgtggtgttctagtttttttgttatactgaaataaatttt
cagagaatgtgggggaaggcggtaattagattaattcaagacgtaattcagaacttagttggccaagctaacgaaatcacccc
aatttatcagtttgattgggaaacttatatattggcgactaaaaaatatgaacgtcatttagaggtgtgtctattagtagaaa
attcgaattgtttttcggattcaaagataatgtgtcaataaaagagatatgaaaggctataattccaaccttatggtaaaggg
ctaggtgt

Isolate 99
cgaatatggtataatattagtaaaagcacagtaataacaaggaatcatcgaaactgaagtcctgctgagacgaatggcgcgat
tacgaaagctcaaaagaaaattttctacgagGTTTTAGAGCTGTGCTGTTTCGAATGGTTCCAAAACacaaaatcttttgttg
ctcctggacgtattGTTTTAGAGCTGTGCTGTTTCGAATGGTTCCAAAACatgtaaggtattgtaaaacttcttcttgcgGTT
TTAGAGCTGTGCTGTTTCGAATGGTTCCAAAACactgttcctataattaaaataaaagaggtaGTTTTAGAGCTGTGCTGTTT
CGAATGGTTCCAAAACtgttccagtaaaaagtaattttaaagcattGTTTTAGAGCTGTGCTGTTTCGAATGGTTCCAAAACc
gctcgattgatgctatcaactatattgaaGTTTTAGAGCTGTGCTGTTTCGAATGGTTCCAAAACttcttcaagagaacttgt
agaacagcttcaGTTTTAGAGCTGTGCTGTTTCGAATGGTTCCAAAACaaggtacttttagcttgttcttgtggtgttGTTTT
AGAGCTGTGCTGTTTCGAATGGTTCCAAAACacagctactgtaaattctgcttttacggttGTTTTAGAGCTGTGCTGTTTCG
AATGGTTCCAAAACtagtgcagttgtcaaggagattgtgagcgaGTTTTAGAGCTGTGCTGTTTCGAATGGTTCCAAAACttt
aacctttgaaaatgtgaaaggctcgtaGTTTTAGAGCTGTGCTGTTTCGAATGGTTCCAAAACgcgatgatggtaagtcatca
tggacagcgtGTTTTAGAGCTGTGCTGTTTCGAATGGTTCCAAAACttgctgctagacccaaacagtttatttttagGTTTTA
GAGCTGTGCTGTTTCGAATGGTTCCAAAACtcctttttttagataatgtgcgatcacggacGTTTTAGAGCTGTGCTGTTTCGA
ATGGTTCCAAAACttttaccaatgcttccatatcgcttatatGTTTTAGAGCTGTGCTGTTTCGAATGGTTCCAAAACtggtt
atacatttactaatccatcagcattGTTTTAGAGCTGTGCTGTTTCGAATGGTTCCAAAACaagctaattctcatctcaccga
gatggataGTTTTAGAGCTGTGCTGTTATTATGCTAGGACATCAttgtggtgttctagtttttttgttatactgaaataaattt
tcagagaatgtgggggaaggcggtaattagattaattcaagacgtaattcagaacttagttggccaagctaacgaaatcaccc
caatttatcagtttgattgggaaacttatatattggcgactaaaaaatatgaacgtcatttagaggtgtgtctattagtagaa

aattcgaattgtttttcggattcaaagataatgtgtcaataaaagagatatgaaaggctataattccaaccttatggtaaagg
gctaggtgtt

Woman 21

Isolate 104

Tatggtataatattagttaaaagcacagtaataacaaggaatcactcgaaactgaagtcctgctgagacgaatggcgcgatta
cgaaagctcaaaagaaaattttctacgaggttttagagctgtgctgtttcgaatgGTTTTAGAGCTGTGCTGTTTCGAATGGT
TCCAAAACccgtgcaagagttaaggaactgacacagcaGTTTTAGAGCTGTGCTGTTTCGAATGGTTCCAAAACcatactagc
tttcatgactttcgaacctttGTTTTAGAGCTGTGCTGTTTCGAATGGTTCCAAAACaacacagcttcctcgaaagggatata
tctaGTTTTAGAGCTGTGCTGTTTCGAATGGTTCCAAAACaatattgaaatagtttgtgcagatggttttGTTTTAGAGCTGT
GCTGTTTCGAATGGTTCCAAAACtcctcgccgtcgacataaccagccatttcaGTTTTAGAGCTGTGCTGTTTCGAATGGTTC
CAAAACaacgatttgcacaataatccaggacaatttGTTTTAGAGCTGTGCTGTTTCGAATGGTTCCAAAACaataggcttag
gcgcatctggttctttactGTTTTAGAGCTGTGCTGTTTCGAATGGTTCCAAAACactataagtatttgttttatccctagt
agGTTTTAGAGCTGTGCTGTTTCGAATGGTTCCAAAACtatctacgagacgaaaacaattgccaatgtGTTTTAGAGCTGTGC
TGTTTCGAATGGTTCCAAAACagttatcaatgctatatggtttctcatcttGTTTTAGAGCTGTGCTGTTTCGAATGGTTCCA
AAACtatttcatcatagaaaatcctgctagtggtGTTTTAGAGCTGTGCTGTTTCGAATGGTTCCAAAACtggttatacattt
actaatccatcagcattGTTTTAGAGCTGTGCTGTTTCGAATGGTTCCAAAACaagctaattctcatctcaccgagatggata
GTTTTAGAGCTGTGCTGTTATTATGCTAGGACATCAttgtggtgttctagtttttttgttatactgaaataaattttcagagaa
tgtgggggaaggcggtaattagattaattcaagacgtaattcagaacttagttggccaagctaacgaaatcaccccaatttat
cagtttgattgggaaacttatatattggcgactaaaaaatatgaacgtcatttagaggtgtgtctattagtagaaaattcgaa
ttgtttttcggattcaaagagaatgtgtcaataaaagagatatgaaaggctataattccaaccttatggttaaaggggctagg
t

Isolate 106

tgaagtcctgctgagacgaatggcgcgattacgaaagctcaaaagaaaattttctacgagGTTTTAGAGCTGTGCTGTTTCGA
ATGGTTCCAAAACccgtgcaagagttaaggaactgacacagcaGTTTTAGAGCTGTGCTGTTTCGAATGGTTCCAAAACcata
ctagctttcatgactttcgaacctttGTTTTAGAGCTGTGCTGTTTCGAATGGTTCCAAAACaacacagcttcctcgaaaggg
atatatctaGTTTTAGAGCTGTGCTGTTTCGAATGGTTCCAAAACaatattgaaatagtttgtgcagatggttttGTTTTAGA
GCTGTGCTGTTTCGAATGGTTCCAAAACtcctcgccgtcgacataaccagccatttcaGTTTTAGAGCTGTGCTGTTTCGAAT
GGTTCCAAAACaacgatttgcacaataatccaggacaatttGTTTTAGAGCTGTGCTGTTTCGAATGGTTCCAAAACaagtgt
gttatcatatcttcctttggttttGTTTTAGAGCTGTGCTGTTTCGAATGGTTCCAAAACaataggcttaggcgcatctggtt
ctttactGTTTTAGAGCTGTGCTGTTTCGAATGGTTCCAAAACactataagtatttgttttatccctagtagGTTTTAGAGC
TGTGCTGTTTCGAATGGTTCCAAAACtatCtacgagacgaaaacaattgccaatgtGTTTTAGAGCTGTGCTGTTTCGAATGG
TTCCAAAACagttatcaatgctatatggtttctcatcttGTTTTAGAGCTGTGCTGTTTCGAATGGTTCCAAAACtatttcat
catagaaaatcctgctagtggtGTTTTAGAGCTGTGCTGTTTCGAATGGTTCCAAAACtggttatacatttactaatccatca
gcattGTTTTAGAGCTGTGCTGTTTCGAATGGTTCCAAAACaagctaattctcatctcaccgagatggataGTTTTAGAGCTG
TGCTGTTATTATGCTAGGACATCAttgtggtgttctagtttttttgttatactgaaataaattttcagagaatgtgggggaagg
cggtaattagattaattcaagacgtaattcagaacttagttggccaagctaacgaaatcaccccaatttatcagtttgattgg
gaaacttatatattggcgactaaaaaatatgaacgtcatttagaggtgtgtctattagtagaaaattcgaattgtttttcgga
ttcaaagagaatgtgtcaataaaagagatatgaaaggctataattccaaccttatggtaaagggctaggt

Woman 30

Isolate 142

Atatggtataatattagtaaaagcacagtaataacaaggaatcatcgaaactgaagtcctgctgagacgaatggcgcgattac
gaaagctcaaaagaaaattttctacgaGTTTTAGAGCTGTGCTGTTTCGAATGGTTCCAAAACtaatttccaatacttacgat
atcttcgtcaGTTTTAGAGCTGTGCTGTTTCGAATGGTTCCAAAACcatgcctttgaaaataaattccgagccattGTTTTAG
AGCTGTGCTGTTTCGAATGGTTCCAAAACacttcattgatgttttgttcaaatttctcaGTTTTAGAGCTGTGCTGTTTCGAA
TGGTTCCAAAACcaaattatagctaagtacccaaacagtattGTTTTAGAGCTGTGCTGTTTCGAATGGTTCCAAAACttgtt
tagcaacatagtctaatgttgcattGTTTTAGAGCTGTGCTGTTTCGAATGGTTCCAAAACtaaagatacacctcaaaatgat
acatctgtGTTTTAGAGCTGTGCTGTTTCGAATGGTTCCAAAACtgttgcttgttcgtattcgtcaggagaagaGTTTTAGAG
CTGTGCTGTTTCGAATGGTTCCAAAACcaaccctatgtttgataatattttagacgtGTTTTAGAGCTGTGCTGTTTCGAATG
GTTCCAAAACaagtgaagttgaattttatttgagatactaGTTTTAGAGCTGTGCTGTTTCGAATGGTTCCAAAACtaatact
tttacaatatgtgttttcactacGTTTTAGAGCTGTGCTGTTTCGAATGGTTCCAAAACaagtgccacagtttgtggctgatt
ggattgGTTTTAGAGCTGTGCTGTTTCGAATGGTTCCAAAACcattcaaggactaccctcaacagtaactctGTTTTAGAGCT
GTGCTGTTTCGAATGGTTCCAAAACataatatattatataataaatatagaaataGTTTTAGAGCTGTGCTGTTTCGAATGGT
TCCAAAACaaataatattgattttcaaaattattttaaGTTTTAGAGCTGTGCTGTTTCGAATGGTTCCAAAACttagcagaa
ttaagtatgtattatatgaaGTTTTAGAGCTGTGCTGTTTCGAATGGTTCCAAAACctcaaaacctttaattaagatagtagc
attGTTTTAGAGCTGTGCTGTTTCGAATGGTTCCAAAACtcttctttttaattcttctaacactccatcGTTTTAGAGCTGTG
CTGTTTCGAATGGTTCCAAAACatcttcttttgacctaacaaaaggatatgtGTTTTAGAGCTGTGCTGTTATTATGCTAGGA
CATCAttgtggtgttctagtttttttgttatactgaaataaattttcagagaatgtgggggaaggcggtaattagattaattca
agacgtaattcagaacttagttggccaagctaacgaaatcaccccaatttatcagtttgattgggaaacttatatattggcga
ctaaaaaatatgaacgtcatttagaggtgtgtctattagtagaaaattcgaattgtttttcggattcaaagagaatgtgtcaa
taaaagagatatgaaaggctataattccaaccttatggtaaagggctaggtgtctacgc

Isolate 143

atatggtataatattagtaaaagcacagtaataacaaggaatcatcgaaactgaagtcctgctgagacgaatggcgcgattac
gaaagctcaaaagaaaattttctacgaGTTTTAGAGCTGTGCTGTTTCGAATGGTTCCAAAACtaatttccaatacttacgat

atcttcgtcaGTTTTAGAGCTGTGCTGTTTCGAATGGTTCCAAAACcatgcctttgaaaataaattccgagccattGTTTTAG
AGCTGTGCTGTTTCGAATGGTTCCAAAACacttcattgatgttttgttcaaatttctcaGTTTTAGAGCTGTGCTGTTTCGAA
TGGTTCCAAAACcaaattatagctaagtacccaaacagtattGTTTTAGAGCTGTGCTGTTTCGAATGGTTCCAAAACttgtt
tagcaacatagtctaatgttgcattGTTTTAGAGCTGTGCTGTTTCGAATGGTTCCAAAACtaaagatacacctcaaaatgat
acatctgtGTTTTAGAGCTGTGCTGTTTCGAATGGTTCCAAAACtgttgcttgttcgtattcgtcaggagaagaGTTTTAGAG
CTGTGCTGTTTCGAATGGTTCCAAAACcaaccctatgtttgataatatttagacgtGTTTTAGAGCTGTGCTGTTTCGAATG
GTTCCAAAACaagtgaagttgaattttatttgagatactaGTTTTAGAGCTGTGCTGTTTCGAATGGTTCCAAAACtaatact
tttacaatatgtgttttcactacGTTTTAGAGCTGTGCTGTTTCGAATGGTTCCAAAACaagtgccacagtttgtggctgatt
ggattgGTTTTAGAGCTGTGCTGTTTCGAATGGTTCCAAAACcattcaaggactaccctcaacagtaactctGTTTTAGAGCT
GTGCTGTTTCGAATGGTTCCAAAACataatatattatataataaatatagaaataGTTTTAGAGCTGTGCTGTTTCGAATGGT
TCCAAAACaaataatattgattttcaaaattattttaaGTTTTAGAGCTGTGCTGTTTCGAATGGTTCCAAAACttagcagaa
ttaagtatgtattatatgaaGTTTTAGAGCTGTGCTGTTTCGAATGGTTCCAAAACatcttcttttgacctaacaaaaggata
tgtGTTTTAGAGCTGTGCTGTTATTATGCTAGGACATCAttgtggtgttctagtttttttgttatactgaaataaattttcaga
gaatgtgggggaaggcggtaattagattaattcaagacgtaattcagaacttagttggccaagctaacgaaatcaccccaatt
tatcagtttgattgggaaacttatatattggcgactaaaaaatatgaacgtcatttagaggtgtgtctattagtagaaaattc
gaattgtttttcggattcaaagagaatgtgtcaataaaagagatatgaaaggctataattccaaccttatggtaaagggctag
g

Woman 42

Isolate 231

aaactgaagtcctgctgagacgaatggcgcgattacgaaagctcaaaagaaaattttctacgagGTTTTAGAGCTGTGCTGTT
TCGAATGGTTCCAAAACgactgcacaacgacaatgcgggtgaaatggGTTTTAGAGCTGTGCTGTTTCGAATGGTTCCAAAAC
tgatacctttattttcatacacaatccactGTTTTAGAGCTGTGCTGTTTCGAATGGTTCCAAAACgaagtatactacggtta
gagattggctcaaGTTTTAGAGCTGTGCTGTTTCGAATGGTTCCAAAACgaaacttcgattagtttgcgtactcgctcaGTTT
TAGAGCTGTGCTGTTTCGAATGGTTCCAAAACAttagtcctgttgttatgatggatgattatGTTTTAGAGCTGTGCTGTTTC
GAATGGTTCCAAAACacaaacctctaatggataatatagaacaaaGTTTTAGAGCTGTGCTGTTTCGAATGGTTCCAAAACac
acaattaaacataaagaagtgcttatttGTTTTAGAGCTGTGCTGTTTCGAATGGTTCCAAAACtcaggagattgtgtgtact
cacgaattttGTTTTAGAGCTGTGCTGTTTCGAATGGTTCCAAAACaaattgtttttgttgtaatataaagtcgacGTTTTA
GAGCTGTGCTGTTTCGAATGGTTCCAAAACaaccttgataggcgtactgtatggattccaGTTTTAGAGCTGTGCTGTTTCGA
ATGGTTCCAAAACtctcgggaaatgcaactgatgctgcaatcaGTTTTAGAGCTGTGCTGTTTCGAATGGTTCCAAAACaaat
tgtttttgttgtaatataaagtcgacGTTTTAGAGCTGTGCTGTTTCGAATGGTTCCAAAACgctagcatggcacaaaaatag
cgttggaatGTTTTAGAGCTGTGCTGTTTCGAATGGTTCCAAAACttaataactgtccgttttgccatttcttgcGTTTTAGA
GCTGTGCTGTTTCGAATGGTTCCAAAACgatggtacaaaatcatttgttggtactgatGTTTTAAAGCTGTGCTGTTATTATG
CTAGGGCACCAttgtggtgttctagtttttttgttatactgaaataaattttcagagaatgtgggggaaggcggtaattagatt
aattcaagacgtaattcagaacttagttggccaagctaacgaaatcaccccaatttatcagtttgattgggaaacttatatat
tggcgactaaaaaatatgaacgtcatttagaggtgttctattagtagaaaattggaattttt

Isolate 232

atatggtataatattagtaaaagcacagtaataacaaggaatcatcgaaactgaagtcctgctgagacgaatggcgcgattac
gaaagctcaaaagaaaattttctacgagGTTTTAGAGCTGTGCTGTTTCGAATGGTTCCAAAACgactgcacaacgacaatgc
gggtgaaatggGTTTTAGAGCTGTGCTGTTTCGAATGGTTCCAAAACtgatacctttattttcatacacaatccactGTTTTA
GAGCTGTGCTGTTTCGAATGGTTCCAAAACgaagtatactacggttagagattggctcaaGTTTTAGAGCTGTGCTGTTTCGA
ATGGTTCCAAAACgaaacttcgattagtttgcgtactcgctcaGTTTTAGAGCTGTGCTGTTTCGAATGGTTCCAAAACAtta
gtcctgttgttatgatggatgattatGTTTTAGAGCTGTGCTGTTTCGAATGGTTCCAAAACacaaacctctaatggataata
tagaacaaaGTTTTAGAGCTGTGCTGTTTCGAATGGTTCCAAAACacacaattaaacataaagaagtgcttatttGTTTTAGA
GCTGTGCTGTTTCGAATGGTTCCAAAACtcaggagattgtgtgtactcacgaattttGTTTTAGAGCTGTGCTGTTTCGAAT
GGTTCCAAAACaaattgtttttgttgtaatataaagtcgacGTTTTAGAGCTGTGCTGTTTCGAATGGTTCCAAAACaacctt
gataggcgtactgtatggattccaGTTTTAGAGCTGTGCTGTTTCGAATGGTTCCAAAACtctcgggaaatgcaactgatgct
gcaatcaGTTTTAGAGCTGTGCTGTTTCGAATGGTTCCAAAACaaattgtttttgttgtaatataaagtcgacGTTTTAGAGC
TGTGCTGTTTCGAATGGTTCCAAAACgctagcatggcacaaaaatagcgttggaatGTTTTAGAGCTGTGCTGTTTCGAATGG
TTCCAAAACtcaggagattgtgtgtactcacgaattttGTTTTAGAGCTGTGCTGTTTCGAATGGTTCCAAAACaaattgtt
tttgttgtaatataaagtcgacGTTTTAGAGCTGTGCTGTTTCGAATGGTTCCAAAACgatggtacaaaatcatttgttggta
ctgatGTTTTAAAGCTGTGCTGTTATTATGCTAGGGCACCAttgtggtgttctagtttttttgttatactgaaataaattttca
gagaatgtgggggaaggcggtaattagattaattcaagacgtaattcagaacttagttggccaagctaacgaaatcaccccaa
tttatcagtttgattgggaaacttatatattggcgactaaaaaatatgaacgtcatttagaggtgtgtctattagtagaaaat
tcgaattgtttttcggattcaaagagaatgtgtcgataaaagagatatgaaaggctataattccaaccttatggttaaagggc
aagatt

Woman 46

Isolate 249

tttcttgataggcgaaatatggtataatattagtaaaagcacagtaataacaaggaatcatcgaaactgaagtcctgctgaga
cgaatggcgcgattacgaaagctcaaaagaaaattttctacgagGTTTTAGAGCTGTGCTGTTTCGAATGGTTCCAAAACctt
gacgttgttcttgacgatgattgccgtcGTTTTAGAGCTGTGCTGTTTCGAATGGTTCCAAAACaaactgcaacatacttaat
catttttttctGTTTTAGAGCTGTGCTGTTTCGAATGGTTCCAAAACtggaactgaatatcactcgctttttacatcGTTTTA
GAGCTGTGCTGTTTCGAATGGTTCCAAAACaatcgtcttcattgttgcgatgattttgatGTTTTAGAGCTGTGCTGTTTCGA
ATGGTTCCAAAACaacattagccttttctaactcttcagctgtGTTTTAGAGCTGTGCTGTTTCGAATGGTTCCAAAACtatg
tcttctaacagttgcttcttgtgcttGTTTTAGAGCTGTGCTGTTTCGAATGGTTCCAAAACccgtcaaacaagagcgacagc

gaaacaagcGTTTTAGAGCTGTGCTGTTTCGAATGGTTCCAAAACttaacagtttcaagtctgtcttgttacttaGTTTTAGA
GCTGTGCTGTTTCGAATGGTTCCAAAACACTCTAAATGATAGTTATGAGTTAAATGTTGTTTTAGAGCTGTGCTGTTTCGAAT
GGTTCCAAAACCaaattacagtttcgactgattatggaaatGTTTTAGAGCTGTGCTGTTTCGAATGGTTCCAAAACatatgt
tccactctatgaatttaggctcatGTTTTAGAGCTGTGCTGTTTCGAATGGTTCCAAAACtttttaccaatgcttccatatcg
cttatatGTTTTAGAGCTGTGCTGTTTCGAATGGTTCCAAAACtacttgacgaattgaagatgacggaatttaGTTTTAGAGC
TGTGCTGTTTCGAATGGTTCCAAAACaagctaattctcatctcaccgagatggataGTTTTAGAGCTGTGCTGTTATTATGCT
AGGACATCAttgtggtgttctagtttttttgttatactgaaataaattttcagagaatgtgggggaaggcggtaattagattaa
ttcaagacgtaattcagaacttagttggccaagctaacgaaatcaccccaatttatcagtttgattgggaaacttatatattg
gcgactaaaaaatatgaacgtcatttagaggtgtgtctattagtagaaaattcgaattgtttttcggattcaaagagaatgtg
tcaataaaagagatatgaaaggctataattccaaccttatggttaaagggctaggtgtcta

Isolate 254

ttgaaggcgaaatatggtataatattagtaaaagcacagtaataacaaggaatcatcgaaactgaagtcctgctgagacgaat
ggcgcgattacgaaagctcaaaagaaaattttctacgagGTTTTAGAGCTGTGCTGTTTCGAATGGTTCCAAAACcttgacgt
tgttcttgacgatgattgccgtcGTTTTAGAGCTGTGCTGTTTCGAATGGTTCCAAAACaaactgcaacatacttaatcattt
ttttctGTTTTAGAGCTGTGCTGTTTCGAATGGTTCCAAAACtggaactgaatatcactcgcttttttacatcGTTTTAGAGCT
GTGCTGTTTCGAATGGTTCCAAAACaatcgtcttcattgttgcgatgattttgatGTTTTAGAGCTGTGCTGTTTCGAATGGT
TCCAAAACtatgtcttctaacagttgcttcttgtgcttGTTTTAGAGCTGTGCTGTTTCGAATGGTTCCAAAACccgtcaaac
aagagcgacagcgaaacaagcGTTTTAGAGCTGTGCTGTTTCGAATGGTTCCAAAACttaacagtttcaagtctgtcttgtta
cttaGTTTTAGAGCTGTGCTGTTTCGAATGGTTCCAAAACactctaaatgatagttATGAGTTAAATGTTGTTTTAGAGCTGT
GCTGTTTCGAATGGTTCCAAAACcaaattacagtttcgactgattatggaaatGTTTTAGAGCTGTGCTGTTTCGAATGGTTC
CAAAACAtatgttccactctatgaatttaggctcatGTTTTAGAGCTGTGCTGTTTCGAATGGTTCCAAAACtttttaccaat
gcttccatatcgcttatatGTTTTAGAGCTGTGCTGTTTCGAATGGTTCCAAAACtacttgacgaattgaagatgacggaatt
taGTTTTAGAGCTGTGCTGTTTCGAATGGTTCCAAAACaagctaattctcatctcaccgagatggataGTTTTAGAGCTGTGC
TGTTATTATGCTAGGACATCAttgtggtgttctagtttttttgttatactgaaataaattttcagagaatgtgggggaaggcgg
taattagattaattcaagacgtaattcagaacttagttggccaagctaacgaaatcaccccaatttatcagtttgattgggaa
acttatatattggcgactaaaaaatatgaacgtcatttagaggtgtgtctattagtagaaaattcgaattgtttttcggattc
aaagagaatgtgtcaataaaagagatatgaaaggctataattccaaccttatggttaaagggctaggt

Woman 51

Isolate 286

tgaaggcgaatatggtataatattagtaaaagcacagtaataacaaggaatcatcgaaactgaagtcctgctgagacgaatgg
cgcgattacgaaagctcaaaagaaaattttctacgagGTTTTAGAGCTGTGCTGTTTCGAATGGTTCCAAAACcatttgaaac
gctcttccaacgtcgcttctGTTTTAGAGCTGTGCTGTTTCGAATGGTTCCAAAACaatttaaaggtagttttgcaggtgttt
tatGTTTTAGAGCTGTGCTGTTTCGAATGGTTCCAAAACaaaggagctggttttgactatttccaatcgGTTTTAGAGCTGTG
CTGTTTCGAATGGTTCCAAAACagctaagaaaaaaataattttaggaatttaGTTTTAGAGCTGTGCTGTTTCGAATGGTTCC
AAAACttccattttatttactcctcgtctggtattGTTTTAGAGCTGTGCTGTTTCGAATGGTTCCAAAACtggttaaataag
caatcaaagcaggcacacGTTTTAGAGCTGTGCTGTTTCGAATGGTTCCAAAACtatccttttctgagggcgtcggtatttgt
tGTTTTAGAGCTGTGCTGTTTCGAATGGTTCCAAAACatgccaaccctcaatttacttggtcacaaaGTTTTAGAGCTGTGCT
GTTTCGAATGGTTCCAAAACttctatcttctgaagatatttcacaagtgaGTTTTAGAGCTGTGCTGTTTCGAATGGTTCCAA
AACatcttcttttgacctaacaaaaggatatgtGTTTTAGAGCTGTGCTGTTATTATGCTAGGACATCAttgtggtgttctag
tttttttgttatactgaaataaattttcagagaatgtgggggaaggcggtaattagattaattcaagacgtaattcagaactta
gttggccaagctaacgaaatcaccccaatttatcagtttgattgggaaacttatatattggcgactaaaaaatatgaacgtca
tttagaggtgtgtctattagtagaaaattcgaattgtttttcggattcaaagagaatgtgtcaataaaagagatatgaaaggc
tataattccaaccttatggttaaagggctaggtgtcta

Isolate 287

attgcttgataggcgaaatatggtataatattagtaaaagcacagtaataacaaggaatcatcgaaactgaagtcctgctgag
acgaatggcgcgattacgaaagctcaaaagaaaattttctacgagGTTTTAGAGCTGTGCTGTTTCGAATGGTTCCAAAACaa
tttaaaggtagttttgcaggtgttttatGTTTTAGAGCTGTGCTGTTTCGAATGGTTCCAAAACaaaggagctggttttgact
atttccaatcgGTTTTAGAGCTGTGCTGTTTCGAATGGTTCCAAAACagctaagaaaaaaataattttaggaatttaGTTTTA
GAGCTGTGCTGTTTCGAATGGTTCCAAAACttccattttatttactcctcgtctggtattGTTTTAGAGCTGTGCTGTTTCGA
ATGGTTCCAAAACtggttaaataagcaatcaaagcaggcacacGTTTTAGAGCTGTGCTGTTTCGAATGGTTCCAAAACtatc
cttttctgagggcgtcggtatttgttGTTTTAGAGCTGTGCTGTTTCGAATGGTTCCAAAACatgccaaccctcaatttactt
ggtcacaaaGTTTTAGAGCTGTGCTGTTTCGAATGGTTCCAAAACttctatcttctgaagatatttcacaagtgaGTTTTAGA
GCTGTGCTGTTTCGAATGGTTCCAAAACatcttcttttgacctaacaaaaggatatgtGTTTTAGAGCTGTGCTGTTATTATG
CTAGGACATCAttgtggtgttctagtttttttgttatactgaaataaattttcagagaatgtgggggaaggcggtaattagatt
aattcagacgtaatttcagaacttagttggccagctaacgaatcacccatttatcagttgatggaac

Woman 63

Isolate 357

Caaagggatagggttattgctttgataggcgaaaatatggtataatattagtaaaagcacagtaataacaaggaatcatcgaa
actgaagtcctgctgagacgaatggcgcgattacgaaagctcaaaagaaaattttctacgagGTTTTAGAGCTGTGCTGTTTC
GAATGGTTCCAAAACagtcaatgctgttaagacagcatttcaaggGTTTTAGAGCTGTGCTGTTTCGAATGGTTCCAAAACct
ggagttgctaaagtacctcgtccttggGTTTTAGAGCTGTGCTGTTTCGAATGGTTCCAAAACcaagggctgaaaaacaagcg
atatatcattGTTTTAGAGCTGTGCTGTTTCGAATGGTTCCAAAACacctaaatagggttcataaaagttattattGTTTTAG

AGCTGTGCTGTTTCGAATGGTTCCAAAACttagaagcttatttacaggcgaaaaaaccGTTTTAGAGCTGTGCTGTTTCGAA
TGGTTCCAAAACagctaagaaaaaaataattttaggaatttaGTTTTAGAGCTGTGCTGTTTCGAATGGTTCCAAAACttttc
accgcttcagcaacgtcataaggctGTTTTAGAGCTGTGCTGTTTCGAATGGTTCCAAAACattgacaggtgataccttaaca
ataaaaaaGTTTTAGAGCTGTGCTGTTTCGAATGGTTCCAAAACttctatcttctgaagatatttcacaagtgaGTTTTAGAG
CTGTGCTGTTTCGAATGGTTCCAAAACtcctctttctatgtttcaattgccaattttGTTTTAGAGCTGTGCTGTTATTATGC
TAGGACATCAttgtggtgttctagtttttttgttatactgaaataaattttcagagaatgtgggggaaggcggtaattagatta
attcaagacgtaattcagaacttagttggccaagctaacgaaatcaccccaatttatcagtttgattgggaaacttatatatt
ggcgactaaaaaatatgaacgtcatttagaggtgtgtctattagtagaaaattcgaattgtttttcggattcaaagagaatgt
gtcaataaaagagatatgaaaggctataattccaaccttatggttaaagggctaggttgttcta

Isolate 358

tatggtataatattagtaaaagcacagtaataacaaggaatcatcgaaactgaagtcctgctgagacgaatggcgcgattacg
aaagctcaaaagaaaattttctacgagGTTTTAGAGCTGTGCTGTTTCGAATGGTTCCAAAACctggagttgctaaagtacct
cgtccttggGTTTTAGAGCTGTGCTGTTTCGAATGGTTCCAAAACcaagggctgaaaaacaagcgatatatcattGTTTTAGA
GCTGTGCTGTTTCGAATGGTTCCAAAACacctaaatagggttcataaaagttattattGTTTTAGAGCTGTGCTGTTTCGAAT
GGTTCCAAAACttagaagcttatttacaggcgaaaaaaccGTTTTAGAGCTGTGCTGTTTCGAATGGTTCCAAAACagctaa
gaaaaaaataattttaggaatttaGTTTTAGAGCTGTGCTGTTTCGAATGGTTCCAAAACttttcaccgcttcagcaacgtca
taaggctGTTTTAGAGCTGTGCTGTTTCGAATGGTTCCAAAACattgacaggtgataccttaacaataaaaaaGTTTTAGAGC
TGTGCTGTTTCGAATGGTTCCAAAACttctatcttctgaagatatttcacaagtgaGTTTTAGAGCTGTGCTGTTTCGAATGG
TTCCAAAACtcctctttctatgtttcaattgccaattttGTTTTAGAGCTGTGCTGTTATTATGCTAGGACATCAttgtggtg
ttctagtttttttgttatactgaaataaattttcagagaatgtgggggaaggcggtaattagattaattcaagacgtaattcag
aacttagttggccaagctaacgaaatcaccccaatttatcagtttgattgggaaacttatatattggcgactaaaaaatatga
acgtcatttagaggtgtgtctattagtagaaaattcgaattgtttttcggattcaaagagaatgtgtcaataaaagagatatg
aaaggctataattccaaccttatggtaaagggctaggtgtctac

Woman 67

Isolate 380

gtataatattagtaaaagcacagtaataacaaggaatcatcgaaactgaagtcctgctgagacgaatggcgcgattacgaaag
ctcaaaagaaaattttctacgagGTTTTAGAGCTGTGCTGTTTCGAATGGTTCCAAAACtatcggaacgagtggagtggtaaa
acatttGTTTTAGAGCTGTGCTGTTTCGAATGGTTCCAAAACaaaaatactgtaaaatcgtaatctacataaGTTTTAGAGCT
GTGCTGTTTCGAATGGTTCCAAAACaaatgttattgatgagacaacattaaaattGTTTTAGAGCTGTGCTGTTTCGAATGGT
TCCAAAACtgtgcgtataaatgatagcttgctttccttGTTTTAGAGCTGTGCTGTTTCGAATGGTTCCAAAACatagtcctt
gtgatagaagttggtgtaagaGTTTTAGAGCTGTGCTGTTTCGAATGGTTCCAAAACcaatttctttgctcttgatgatgacg
gtgtGTTTTAGAGCTGTGCTGTTTCGAATGGTTCCAAAACcgttctgctttagtcatatgtgtcctttctGTTTTAGAGCTGT
GCTGTTTCGAATGGTTCCAAAACctactgtattatctgaaatagtatcgttttGTTTTAGAGCTGTGCTGTTTCGAATGGTTC
CAAAACtctcgggaaatgcaactgatgctgcaatcaGTTTTAGAGCTGTGCTGTTTCGAATGGTTCCAAAACaaattgttttt
gttgtaatataaagtcgacGTTTTAGAGCTGTGCTGTTTCGAATGGTTCCAAAACttaataactgtccgttttgccatttctt
gcGTTTTAGAGCTGTGCTGTTTCGAATGGTTCCAAAACagaggggaaaatatcaatgccgaatgctgaGTTTTAGAGCTGTGC
TGTTTCGAATGGTTCCAAAACgatggtacaaaatcatttgttggtactgatGTTTTAAAGCTGTGCTGTTATTATGCTAGGGC
ACCAttgtggtgttctagtttttttgttatactgaaataaattttcagagaatgtgggggaaggcggtaattagattaattcaa
gacgtaattcagaacttagttggccaagctaacgaaatcaccccaatttatcagtttgattgggaaacttatatattggcgac
taaaaaatatgaacgtcatttagaggtgtgtctattagtagaaaattcgaattgtttttcggattcaaagagaatgtgtcgat
aaaagagatatgaaaggctataattccaacctt

Isolate 381

tattagtaaaagcacagtaataacaaggaatcatcgaaactgaagtcctgctgagacgaatggcgcgattacgaaagctcaaa
agaaaattttctacgagGTTTTAGAGCTGTGCTGTTTCGAATGGTTCCAAAACTatcggaacgagtggagtggtaaaacattt
GTTTTAGAGCTGTGCTGTTTCGAATGGTTCCAAAACaaaaatactgtaaaatcgtaatctacataaGTTTTAGAGCTGTGCTG
TTTCGAATGGTTCCAAAACtgtgcgtataaatgatagcttgctttccttGTTTTAGAGCTGTGCTGTTTCGAATGGTTCCAAA
ACtgtgcgtataaatgatagcttgctttccttGTTTTAGAGCTGTGCTGTTTCGAATGGTTCCAAAACatagtccttgtgata
gaagttggtgtaagaGTTTTAGAGCTGTGCTGTTTCGAATGGTTCCAAAACcaatttctttgctcttgatgatgacggtgtGT
TTTAGAGCTGTGCTGTTTCGAATGGTTCCAAAACcgttctgctttagtcatatgtgtcctttctGTTTTAGAGCTGTGCTGTT
TCGAATGGTTCCAAAACctactgtattatctgaaatagtatcgttttGTTTTAGAGCTGTGCTGTTTCGAATGGTTCCAAAAC
tctcgggaaatgcaactgatgctgcaatcaGTTTTAGAGCTGTGCTGTTTCGAATGGTTCCAAAACaaattgtttttgttgta
atataaagtcgacGTTTTAGAGCTGTGCTGTTTCGAATGGTTCCAAAACttaataactgtccgttttgccatttcttgcGTTT
TAGAGCTGTGCTGTTTCGAATGGTTCCAAAACagaggggaaaatatcaatgccgaatgctgaGTTTTAGAGCTGTGCTGTTTC
GAATGGTTCCAAAACgatggtacaaaatcatttgttggtactgatGTTTTAAAGCTGTGCTGTTATTATGCTAGGGCACCAtt
gtggtgttctagtttttttgttatactgaaataaattttcagagaatgtgggggaaggcggtaattagattaattcaagacgta
attcagaacttagttggccaagctaacgaaatcaccccaatttatcagtttgattgggaaacttatatattggcgactaaaaa
atatgaacgtcatttagaggtgtgtctattagtagaaaattcgaattgtttttcggattcaaagagaatgtgtcgataaaaga
gatatgaaaggctataattccaaccttatggttaaagggcaa

Isolate 382

Gaaagggaaaggattaatttcttgataggcgaaatatggtataatattagtaaaagcacagtaataacaaggaatcatcgaaa
ctgaagtcctgctgagacgaatggcgcgattacgaaagctcaaaagaaaattttctacgagGTTTTAGAGCTGTGCTGTTTCG
AATGGTTCCAAAACtatcggaacgagtggagtggtaaaacatttGTTTTAGAGCTGTGCTGTTTCGAATGGTTCCAAAACaaa
aatactgtaaaatcgtaatctacataaGTTTTAGAGCTGTGCTGTTTCGAATGGTTCCAAAACaaatgttattgatgagacaa

cattaaaattGTTTTAGAGCTGTGCTGTTTCGAATGGTTCCAAAACtgtgcgtataaatgatagcttgctttccttGTTTTAG
AGCTGTGCTGTTTCGAATGGTTCCAAAACatagtccttgtgatagaagttggtgtaagaGTTTTAGAGCTGTGCTGTTTCGAA
TGGTTCCAAAACcaatttctttgctcttgatgatgacggtgtGTTTTAGAGCTGTGCTGTTTCGAATGGTTCCAAAACcgttc
tgctttagtcatatgtgtcctttctGTTTTAGAGCTGTGCTGTTTCGAATGGTTCCAAAACctactgtattatctgaaatagt
atcgttttGTTTTAGAGCTGTGCTGTTTCGAATGGTTCCAAAACtctcgggaaatgcaactgatgctgcaatcaGTTTTAGAG
CTGTGCTGTTTCGAATGGTTCCAAAACaaattgtttttgttgtaatataaagtcgacGTTTTAGAGCTGTGCTGTTTCGAATG
GTTCCAAAACttaataactgtccgttttgccatttcttgcGTTTTAGAGCTGTGCTGTTTCGAATGGTTCCAAAACagaggggg
aaaatatcaatgccgaatgctgaGTTTTAGAGCTGTGCTGTTTCGAATGGTTCCAAAACgatggtacaaaatcatttgttggt
actgatGTTTTAAAGCTGTGCTGTTATTATGCTAGGGCACCAttgtggtgttctagtttttttgttatactgaaataaattttc
agagaatgtgggggaaggcggtaattagattaattcaagacgtaattcagaacttagttggccaagctaacgaaatcacccca
atttatcagtttgattgggaaacttatatattggcgactaaaaaatatgaacgtcatttagaggtgtgtctattagtagaaaa
ttcgaattgtttttcggattcaaagagaatgtgtcgataaaaaagatatgaaaggctataattccaaccttatggttaaagg

Woman 75

Isolate 427

gcgaaatatggtataatattagtaaaagcacagtaataacaaggaatcatcgaaactgaagtcctgctgagacgaatggcgcg
attacgaaagctcaaaagaaaattttctacgagGTTTTAGAGCTGTGCTGTTTCGAATGGTTCCAAAACaaactaaagatatt
gactccctgcaggataGTTTTAGAGCTGTGCTGTTTCGAATGGTTCCAAAACaacattagccttttctaactcttcagctgtG
TTTTAGAGCTGTGCTGTTTCGAATGGTTCCAAAACtatgtcttctaacagttgcttcttgtgcttGTTTTAGAGCTGTGCTGT
TTCGAATGGTTCCAAAACccgtcaaacaagagcgacagcgaaacaagcGTTTTAGAGCTGTGCTGTTTCGAATGGTTCCAAAA
CtttttattggttttctaagtgctcgaccatcGTTTTAGAGCTGTGCTGTTTCGAATGGTTCCAAAACagttacttctgcttgg
gtttgataagggtcGTTTTAGAGCTGTGCTGTTTCGAATGGTTCCAAAACgaaatgtggagtcattcaggttgatgatggGTT
TTAGAGCTGTGCTGTTTCGAATGGTTCCAAAACaaaaaataaatgactttaaagcacttggagGTTTTAGAGCTGTGCTGTTT
CGAATGGTTCCAAAACtagtagccattattattatggcttttatttGTTTTAGAGCTGTGCTGTTTCGAATGGTTCCAAAACa
agctaattctcatctcaccgagatggataGTTTTAGAGCTGTGCTGTTATTATGCTAGGACATCAttgtggtgttctagtttt
ttgttatactgaaataaattttcagagaatgtgggggaaggcggtaattagattaattcaagacgtaattcagaacttagttg
gccaagctaacgaaatcaccccaatttatcagtttgattgggaaacttatatattggcgactaaaaaatatgaacgtcattta
gaggtgtgtctattagtagaaaattcgaattgtttttcggattcaaagagaatgtgtcaataaaagagatatgaaaggctata
attccaaccttatggtaaagggctaggtgtctacgct

Isolate 428

tttcttgataggcgaaatatggtataatattagtaaaagcacagtaataacaaggaatcatcgaaactgaagtcctgctgaga
cgaatggcgcgattacgaaagctcaaaagaaaattttctacgagGTTTTAGAGCTGTGCTGTTTCGAATGGTTCCAAAACaat
atattcgagcaatcaacaaagggattgGTTTTAGAGCTGTGCTGTTTCGAATGGTTCCAAAACttactcatttattactcctc
tgtgctatgaGTTTTAGAGCTGTGCTGTTTCGAATGGTTCCAAAACtccttatcagataccgaaaatacaaagttaGTTTTAG
AGCTGTGCTGTTTCGAATGGTTCCAAAACaaactaaagatattgactccctgcaggataGTTTTAGAGCTGTGCTGTTTCGAA
TGGTTCCAAAACaacattagccttttctaactcttcagctgtGTTTTAGAGCTGTGCTGTTTCGAATGGTTCCAAAACtatgt
cttctaacagttgcttcttgtgcttGTTTTAGAGCTGTGCTGTTTCGAATGGTTCCAAAACccgtcaaacaagagcgacagcg
aaacaagcGTTTTAGAGCTGTGCTGTTTCGAATGGTTCCAAAACTttattggttttctaagtgctcgaccatcGTTTTAGAG
CTGTGCTGTTTCGAATGGTTCCAAAACagttacttctgcttgggtttgataagggtcGTTTTAGAGCTGTGCTGTTTCGAATG
GTTCCAAAACgaaatgtggagtcattcaggttgatgatggGTTTTAGAGCTGTGCTGTTTCGAATGGTTCCAAAACaaaaaat
aaatgactttaaagcacttggagGTTTTAGAGCTGTGCTGTTTCGAATGGTTCCAAAACtagtagccattattattatggctt
ttatttGTTTTAGAGCTGTGCTGTTTCGAATGGTTCCAAAACaagctaattctcatctcaccgagatggataGTTTTAGAGCT
GTGCTGTTATTATGCTAGGACATCAttgtggtgttctagtttttttgttatactgaaataaattttcagagaatgtgggggaag
gcggtaattagattaattcaagacgtaattcagaacttagttggccaagctaacgaaatcaccccaatttatcagtttgattg
ggaaacttatatattggcgactaaaaaatatgaacgtcatttagaggtgtgtctattagtagaaaattcgaattgtttttcgg
attcaaagagaatgtgtcaataaaagagatatgaaaggctataattccaaccttatggttaaagggctaggtgtctacgct

Woman 76

Isolate 431

gaaatatggtataatattagtaaaagcacagtaataacaaggaattatcgaaactgaagtcctgctgagacgaatggcgcgat
tacgaaagctcaaaagaaaattttctacgaGTTTTAGAGCTGTGCTGTTTCGAATGGTTCCAAAACctgcattattagctttt
gcagagctttataGTTTTAGAGCTGTGCTGTTTCGAATGGTTCCAAAACtgaattccacgccaccaagtaaacctgtgaGTTT
TAGAGCTGTGCTGTTTCGAATGGTTCCAAAACtaagaattttagatactctacttgaatgctGTTTTAGAGCTGTGCTGTTTC
GAATGGTTCCAAAACgggtccaaatcagcaatctatgctaaaaatGTTTTAGAGCTGTGCTGTTTCGAATGGTTCCAAAACca
accctatgtttgataatattttagacgtGTTTTAGAGCTGTGCTGTTTCGAATGGTTCCAAAACaagtgaagttgaattttat
ttgagatactaGTTTTAGAGCTGTGCTGTTTCGAATGGTTCCAAAACtaatacttttacaatatgtgttttcactacGTTTTA
GAGCTGTGCTGTTTCGAATGGTTCCAAAACaagtgccacagtttgtggctgattggattgGTTTTAGAGCTGTGCTGTTTCGA
ATGGTTCCAAAACcattcaaggactaccctcaacagtaactctGTTTTAGAGCTGTGCTGTTTCGAATGGTTCCAAAACataa
tatattatataataaatatagaaataGTTTTAGAGCTGTGCTGTTTCGAATGGTTCCAAAACttctatcttctgaagatattt
cacaagtgaGTTTTAGAGCTGTGCTGTTTCGAATGGTTCCAAAACatcttcttttgacctaacaaaaggatatgtGTTTTAGA
GCTGTGCTGTTATTATGCTAGGACATCAttgtggtgttctagtttttttgttatactgaaataaattttcagagaatgtggggg
aaggcggtaattagattaattcaagacgtaattcagaacttagttggccaagctaacgaaatcaccccaatttatcagtttga
ttgggaaacttatatattggcgactaaaaaatatgaacgtcatttagaggtgtgtctattagtagaaaattcgaattgttttt
cggattcaaagagaatgtgtcaataaaagagatatgaaaggctataattccaaccttatggttaaaggg

Isolate 432
ttgataggcgaatatggtataatattagtaaaagcacagtaataacaaggaattatcgaaactgaagtcctgctgagacgaat
ggcgcgattacgaaagctcaaaagaaaatttttctacgaGTTTTAGAGCTGTGCTGTTTCGAATGGTTCCAAAACctgcattat
tagcttttgcagagctttataGTTTTAGAGCTGTGCTGTTTCGAATGGTTCCAAAACtgaattccacgccaccaagtaaacct
gtgaGTTTTAGAGCTGTGCTGTTTCGAATGGTTCCAAAACtaagaattttagatactctacttgaatgctGTTTTAGAGCTGT
GCTGTTTCGAATGGTTCCAAAACgggtccaaatcagcaatctatgctaaaaatGTTTTAGAGCTGTGCTGTTTCGAATGGTTC
CAAAACcaaccctatgtttgataatattttagacgtGTTTTAGAGCTGTGCTGTTTCGAATGGTTCCAAAACaagtgaagttg
aattttatttgagatactaGTTTTAGAGCTGTGCTGTTTCGAATGGTTCCAAAACtaatacttttacaatatgtgttttcact
acGTTTTAGAGCTGTGCTGTTTCGAATGGTTCCAAAACaagtgccacagtttgtggctgattggattgGTTTTAGAGCTGTGC
TGTTTCGAATGGTTCCAAAACaagtgccacagtttgtggctgattggattgGTTTTAGAGCTGTGCTGTTTCGAATGGTTCCA
AAACcattcaaggactaccctcaacagtaactctGTTTTAGAGCTGTGCTGTTTCGAATGGTTCCAAAACataatatattata
taataaatatagaaataGTTTTAGAGCTGTGCTGTTTCGAATGGTTCCAAAACttctatcttctgaagatatttcacaagtga
GTTTTAGAGCTGTGCTGTTTCGAATGGTTCCAAAACatcttcttttgacctaacaaaaggatatgtGTTTTAGAGCTGTGCTG
TTATTATGCTAGGACATCAttgtggtgttctagtttttttgttatactgaaataaattttcagagaatgtgggggaaggcggta
attagattaattcaagacgtaattcagaacttagttggccaagctaacgaaatcaccccaatttatcagtttgattgggaaac
ttatatattggcgactaaaaaatatgaacgtcatttagaggtgtgtctattagtagaaaattcgaattgtttttcggattcaa
agagaatgtgtcaataaaagagatatgaaaggctataattccaaccttatggttaaa

Woman 77

Isolate 433
gcgaaatatggtataatattagtaaaagcacagtaataacaaggaatcatcgaaactgaagtcctgctgagacgaatggcgcg
attacgaaagctcaaaagaaaatttttctacgagGTTTTAGAGCTGTGCTGTTTCGAATGGTTCCAAAACtcattcatcttctc
aagttgccagatataaGTTTTAGAGCTGTGCTGTTTCGAATGGTTCCAAAACcaattaactttaataacctactataattctG
TTTTAGAGCTGTGCTGTTTCGAATGGTTCCAAAACaatttctatggaagaaaccgcagcagcaatGTTTTAGAGCTGTGCTGT
TTCGAATGGTTCCAAAACatacaagaatatcactgaactactcaaaaaGTTTTAGAGCTGTGCTGTTTCGAATGGTTCCAAAA
CgaaacttcgattagtttgcgtactcgctcaGTTTTAGAGCTGTGCTGTTTCGAATGGTTCCAAAACattagtcctgttgtta
tgatggatgattatGTTTTAGAGCTGTGCTGTTTCGAATGGTTCCAAAACacaaacctctaatggataatatagaacaaaGTT
TTAGAGCTGTGCTGTTTCGAATGGTTCCAAAACacacaattaaacataaagaagtgcttatttGTTTTAGAGCTGTGCTGTTT
CGAATGGTTCCAAAACttttcagctgaggaagtaatcttttcttctGTTTTAGAGCTGTGCTGTTTCGAATGGTTCCAAAACt
caggagattgtgtgtactcacgaatttttGTTTTAGAGCTGTGCTGTTTCGAATGGTTCCAAAACgaatgaagcaggtatcaa
ggattatacttaGTTTTAGAGCTGTGCTGTTTCGAATGGTTCCAAAACaaattgtttttgttgtaatataaagtcgacGTTTT
AGAGCTGTGCTGTTTCGAATGGTTCCAAAACaaccttgataggcgtactgtatggattccaGTTTTAGAGCTGTGCTGTTTCG
AATGGTTCCAAAACtctcgggaaatgcaactgatgctgcaatcaGTTTTAGAGCTGTGCTGTTTCGAATGGTTCCAAAACtta
ataactgtccgttttgccatttcttgcGTTTTAGAGCTGTGCTGTTTCGAATGGTTCCAAAACagaggggaaaatatcaatgc
cgaatgctgaGTTTTAGAGCTGTGCTGTTTCGAATGGTTCCAAAACgatggtacaaaatcatttgttggtactgatGTTTTAA
AGCTGTGCTGTTATTATGCTAGGGCACCAttgtggtgttctagtttttttgttatactgaaataaattttcagagaatgtgggg
gaaggcggtaattagattaattcaagacgtaattcagaacttagttggccaagctaacgaaatcaccccaatttatcagtttg
attgggaaacttatatattggcgactaaaaaatatgaacgtcatttagaggtgtgtctattagtagaaaattcgaattgtttt
tcggattcaaagagaatgtgtcgataaaatagatatgaaaggctataattccaaccttatggtaaagggcaagatgtctacgc
gggcccaaataaactgaaac

Isolate 433
gcgaatatggtataatattagtaaaagcacagtaataacaaggaatcatcgaaactgaagtcctgctgagacgaatggcgcga
ttacgaaagctcaaaagaaaatttttctacgagGTTTTAGAGCTGTGCTGTTTCGAATGGTTCCAAAACtcattcatcttctca
agttgccagatataaGTTTTAGAGCTGTGCTGTTTCGAATGGTTCCAAAACcaattaactttaataacctactataattctGT
TTTAGAGCTGTGCTGTTTCGAATGGTTCCAAAACaatttctatggaagaaaccgcagcagcaatGTTTTAGAGCTGTGCTGTT
TCGAATGGTTCCAAAACatacaagaatatcactgaactactcaaaaaGTTTTAGAGCTGTGCTGTTTCGAATGGTTCCAAAAC
gaaacttcgattagtttgcgtactcgctcaGTTTTAGAGCTGTGCTGTTTCGAATGGTTCCAAAACattagtcctgttgttat
gatggatgattatGTTTTAGAGCTGTGCTGTTTCGAATGGTTCCAAAACacaaacctctaatggataatatagaacaaaGTTT
TAGAGCTGTGCTGTTTCGAATGGTTCCAAAACacacaattaaacataaagaagtgcttatttGTTTTAGAGCTGTGCTGTTTC
GAATGGTTCCAAAACttttcagctgaggaagtaatcttttcttctGTTTTAGAGCTGTGCTGTTTCGAATGGTTCCAAAACtc
aggagattgtgtgtactcacgaatttttGTTTTAGAGCTGTGCTGTTTCGAATGGTTCCAAAACgaatgaagcaggtatcaag
gattatacttaGTTTTAGAGCTGTGCTGTTTCGAATGGTTCCAAAACaaattgtttttgttgtaatataaagtcgacGTTTTA
GAGCTGTGCTGTTTCGAATGGTTCCAAAACaaccttgataggcgtactgtatggattccaGTTTTAGAGCTGTGCTGTTTCGA
ATGGTTCCAAAACtctcgggaaatgcaactgatgctgcaatcaGTTTTAGAGCTGTGCTGTTTCGAATGGTTCCAAAACaaat
tgtttttgttgtaatataaagtcgacGTTTTAGAGCTGTGCTGTTTCGAATGGTTCCAAAACttaataactgtccgttttgcc
atttcttgcGTTTTAGAGCTGTGCTGTTTCGAATGGTTCCAAAACagaggggaaaatatcaatgccgaatgctgaGTTTTAGA
GCTGTGCTGTTTCGAATGGTTCCAAAACgatggtacaaaatcatttgttggtactgatGTTTTAAAGCTGTGCTGTTATTATG
CTAGGGCACCAttgtggtgttctagtttttttgttatactgaaataaattttcagagaatgtgggggaaggcggtaattagatt
aattcaagacgtaattcagaacttagttggccaagctaacgaaatcaccccaatttatcagtttgattgggaaacttatatat
tggcgactaaaaaatatgaacgtcatttagaggtgtgtctattagtagaaaattcgaattgtttttcggattcaaagagaatg
tgtcgataaaagagatatgaaaggctataattccaaccttatggttaaagggcaagatgtctacgctggaccttaataatagg
aacgc

Woman 79

Isolate 439

ctttcttgataggcgaatatggtataatattagtaaaagcacagtaataacaaggaatcatcgaaactgaagtcctgctgaga
cgaatggcgcgattacgaaagctcaaaagaaaattttctacgaGTTTTAGAGCTGTGCTGTTTCGAATGGTTCCAAAACttgg
aaagatggcagaggggttaacgcagcGTTTTAGAGCTGTGCTGTTTCGAATGGTTCCAAAACttttgagttttcaacttatag
agtgtatttGTTTTAGAGCTGTGCTGTTTCGAATGGTTCCAAAACatctggaagcggtcaagaaagcccagtatgGTTTTAGA
GCTGTGCTGTTTCGAATGGTTCCAAAACacggcacttttaaccgtctctccgcttcttGTTTTAGAGCTGTGCTGTTTCGAAT
GGTTCCAAAACatggtcagaaatcgaagaaaacgaagtcgtGTTTTAGAGCTGTGCTGTTTCGAATGGTTCCAAAACaactaa
acaaacgcattgtgtttttagcttGTTTTAGAGCTGTGCTGTTTCGAATGGTTCCAAAACcctctatcgaggtttcttacggg
tagggtcGTTTTAGAGCTGTGCTGTTTCGAATGGTTCCAAAACgacgaatttagataacaaaaaagccactgtGTTTTAGAGC
TGTGCTGTTTCGAATGGTTCCAAAACatggtcagaaatcgaagaaaacgaagtcgtGTTTTAGAGCTGTGCTGTTTCGAATGG
TTCCAAAACCttatgggagttcttgcaatattagttgtGTTTTAGAGCTGTGCTGTTTCGAATGGTTCCAAAACgtaacccgt
cttttccatcagtcccatcttGTTTTAGAGCTGTGCTGTTTCGAATGGTTCCAAAACttggaaaaaacacgaaagtgatatta
ctttGTTTTAGAGCTGTGCTGTTTCGAATGGTTCCAAAACtaagaattttagatactctacttgaatgctGTTTTAGAGCTGT
GCTGTTTCGAATGGTTCCAAAACaagacttaaaatcgattagaattgatttaGTTTTAGAGCTGTGCTGTTTCGAATGGTTC
CAAAACtcaagatgataatattttaacgcttctaaaGTTTTAGAGCTGTGCTGTTTCGAATGGTTCCAAAACccagtttagaa
acagaacaaaactaacgaaGTTTTAGAGCTGTGCTGTTTCGAATGGTTCCAAAACagacaaagaagatggcaagtctatcaac
aaGTTTTAGAGCTGTGCTGTTTCGAATGGTTCCAAAACagtagaaacataacgataattccatgaataGTTTTAGAGCTGTGC
TGTTTCGAATGGTTCCAAAACatgttgtagaatcatatcgaccatataaccGTTTTAGAGCTGTGCTGTTTCGAATGGTTCCA
AAACcaattgattgccgttaaaaccgatagaggaGTTTTAGAGCTGTGCTGTTTCGAATGGTTCCAAAACatcttcttttgac
ctaacaaaaggatatgtGTTTTAGAGCTGTGCTGTTATTATGCTAGGACATCAttgtggtgttctagtttttttgttatactga
aataaattttcagagaatgtgggggaaggcggtaattagattaattcaagacgtaattcagaacttagttggccaagctaacg
aaatcaccccaatttatcagtttgattgggaaacttatatattggcgactaaaaaatatgaacgtcatttagaggtgtgtcta
ttagtagaaaattcgaattgtttttcggattcaaagagaatgtgtcaataaaagagatatgaaaggctataattccaacctta
tggttaaagggctaggtgtctacgccg

Isolate 440

aggcgaatatggtataatattagtaaaagcacagtaataacaaggaatcatcgaaactgaagtcctgctgagacgaatggcgc
gattacgaaagctcaaaagaaaattttctacgaGTTTTAGAGCTGTGCTGTTTCGAATGGTTCCAAAACttggaaagatggca
gaggggttaacgcagcGTTTTAGAGCTGTGCTGTTTCGAATGGTTCCAAAACttttgagttttcaacttatagagtgtatttG
TTTTAGAGCTGTGCTGTTTCGAATGGTTCCAAAACatctggaagcggtcaagaaagcccagtatgGTTTTAGAGCTGTGCTGT
TTCGAATGGTTCCAAAACacggcacttttaaccgtctctccgcttcttGTTTTAGAGCTGTGCTGTTTCGAATGGTTCCAAAA
CatggtcagaaatcgaagaaaacgaagtcgtGTTTTAGAGCTGTGCTGTTTCGAATGGTTCCAAAACaactaaacaaacgcat
tgtgtttttagcttGTTTTAGAGCTGTGCTGTTTCGAATGGTTCCAAAACcctctatcgaggtttcttacgggtagggtcGTT
TTAGAGCTGTGCTGTTTCGAATGGTTCCAAAACgacgaatttagataacaaaaaagccactgtGTTTTAGAGCTGTGCTGTTT
CGAATGGTTCCAAAACatggtcagaaatcgaagaaaacgaagtcgtGTTTTAGAGCTGTGCTGTTTCGAATGGTTCCAAAACc
gctatctcttgtactggtttcggttctgtGTTTTAGAGCTGTGCTGTTTCGAATGGTTCCAAAACcttatgggagttcttgca
atattagttgtGTTTTAGAGCTGTGCTGTTTCGAATGGTTCCAAAACgtaacccgtcttttccatcagtcccatcttGTTTTA
GAGCTGTGCTGTTTCGAATGGTTCCAAAACttggaaaaaacacgaaagtgatattactttGTTTTAGAGCTGTGCTGTTTCGA
ATGGTTCCAAAACtaagaattttagatactctacttgaatgctGTTTTAGAGCTGTGCTGTTTCGAATGGTTCCAAAACaaga
cttaaaatcgattagaattgatttaGTTTTAGAGCTGTGCTGTTTCGAATGGTTCCAAAACtcaagatgataatattttaac
gcttctaaaGTTTTAGAGCTGTGCTGTTTCGAATGGTTCCAAAACccagtttagaaacagaacaaaactaacgaaGTTTTAGA
GCTGTGCTGTTTCGAATGGTTCCAAAACagacaaagaagatggcaagtctatcaacaaGTTTTAGAGCTGTGCTGTTTCGAAT
GGTTCCAAAACagtagaaacataacgataattccatgaataGTTTTAGAGCTGTGCTGTTTCGAATGGTTCCAAAACatgttg
tagaatcatatcgaccatataaccGTTTTAGAGCTGTGCTGTTTCGAATGGTTCCAAAACcaattgattgccgttaaaaccga
tagaggaGTTTTAGAGCTGTGCTGTTTCGAATGGTTCCAAAACatcttcttttgacctaacaaaaggatatgtGTTTTAGAGC
TGTGCTGTTATTATGCTAGGACATCAttgtggtgttctagtttttttgttatactgaaataaattttcagagaatgtgggggaa
ggcggtaattagattaattcaagacgtaattcagaacttagttggccaagctaacgaaatcaccccaatttatcagtttgatt
gggaaacttatatattggcgactaaaaaatatgaacgtcatttagaggtgtgtctattagtagaaaattcgaattgtttttcg
gattcaaagagaatgtgtcaataaaagagatatgaaaggctataattccaaccttatggtaaagggctaggtgtctacgc

Woman 88

Isolate 476

gaatagtaagctaatttcttgataggcgaaatatggtataatattagtaaaagcacagtaataacaaggaatcatcgaaactg
aagtcctgctgagacgaatggcgcgattacgaaagctcaaaagaaaattttctacgagGTTTTAGAGCTGTGCTGTTTCGAAT
GGTTCCAAAACtatctacgagacgaaaacaattgccaatgtGTTTTAGAGCTGTGCTGTTTCGAATGGTTCCAAAACagttat
caatgctatatggtttctcatcttGTTTTAGAGCTGTGCTGTTTCGAATGGTTCCAAAACgtttaattttgaataatatcatg
tttactcGTTTTAGAGCTGTGCTGTTTCGAATGGTTCCAAAACggcgaacttgtaacgtttgacggtaattttGTTTTAGAGC
TGTGCTGTTTCGAATGGTTCCAAAACtaatgtcttttcctagaatgtacatttctaGTTTTAGAGCTGTGCTGTTTCGAATGG
TTCCAAAACattactttcaagatgtctatgactatatgcGTTTTAGAGCTGTGCTGTTTCGAATGGTTCCAAAACtacttgac
gaattgaagatgacggaatttaGTTTTAGAGCTGTGCTGTTTCGAATGGTTCCAAAACtggttatacatttactaatccatca
gcattGTTTTAGAGCTGTGCTGTTTCGAATGGTTCCAAAACaagctaattctcatctcaccgagatggataGTTTTAGAGCTG
TGCTGTTATTATGCTAGGACATCAttgtggtgttctagtttttttgttatactgaaataaattttcagagaatgtgggggaagg
cggtaattagattaattcaagacgtaattcagaacttagttggccaagctaacgaaatcaccccaatttatcagtttgattgg
gaaacttatatattggcgactaaaaaatatgaacgtcatttagaggtgtgtctattagtagaaaattcgaattgtttttcgga
ttcaaagagaatgtgtcaataaaagagatatgaaaggctataattccaaccttatggttaaagggctaggtgtctacgcttac
taatattttggaccc

Isolate 477

gttaggataagctttttcttgaaggcgaaatatggtataatattagtaaaagcacagtaataacaaggaatcatcgaaactg
aagtcctgctgagacgaatggcgcgattacgaaagctcaaaagaaaattttctacgagGTTTTAGAGCTGTGCTGTTTCGAAT
GGTTCCAAAACtatctacgagacgaaaacaattgccaatgtGTTTTAGAGCTGTGCTGTTTCGAATGGTTCCAAAACagttat
caatgctatatggtttctcatcttGTTTTAGAGCTGTGCTGTTTCGAATGGTTCCAAAACgtttaattttgaataatatcatg
tttactcGTTTTAGAGCTGTGCTGTTTCGAATGGTTCCAAAACgtttaattttgaataatatcatgtttactcGTTTTAGAGC
TGTGCTGTTTCGAATGGTTCCAAAACggcgaacttgtaacgtttgacggtaattttGTTTTAGAGCTGTGCTGTTTCGAATGG
TTCCAAAACtaatgtcttttcctagaatgtacatttctaGTTTTAGAGCTGTGCTGTTTCGAATGGTTCCAAAACattacttt
caagatgtctatgactatatgcGTTTTAGAGCTGTGCTGTTTCGAATGGTTCCAAAACtacttgacgaattgaagatgacgga
atttaGTTTTAGAGCTGTGCTGTTTCGAATGGTTCCAAAACtggttatacatttactaatccatcagcattGTTTTAGAGCTG
TGCTGTTTCGAATGGTTCCAAAACaagctaattctcatctcaccgagatggataGTTTTAGAGCTGTGCTGTTATTATGCTAG
GACATCAttgtggtgttctagtttttttgttatactgaaataaattttcagagaatgtgggggaaggcggtaattagattaatt
caagacgtaattcagaacttagttggccaagctaacgaaatcaccccaatttatcagtttgattgggaaacttatatattggc
gactaaaaaatatgaacgtcatttagaggtgtgtctattagtagaaaattcgaattgtttttcggattcaaagagaatgtgtc
aataaaagagatatgaaaggctataattccaaccttatggttaaagggctaggtgtctacgctttacttaattttttgaacc

Woman 97

Isolate 536

gataggcgaatatggtataatattagtaaaagcacagtaataacaaggaatcatcgaaactgaagtcctgctgagacgaatgg
cgcgattacgaaagctcaaaagaaaattttctacgagGTTTTAGAGCTGTGCTGTTTCGAATGGTTCCAAAACtggaattcaa
agcaaaagcgcttatgaaatGTTTTAGAGCTGTGCTGTTTCGAATGGTTCCAAAACtgctagacttgaaaaagacagttcaga
catGTTTTAGAGCTGTGCTGTTTCGAATGGTTCCAAAACattaaaacctcccattatagctctacccgcGTTTTAGAGCTGTG
CTGTTTCGAATGGTTCCAAAACacaacaatattgtcaataagtaccaacccgGTTTTAGAGCTGTGCTGTTTCGAATGGTTCC
AAAACtaataactaaaaattccacgcttgatttagGTTTTAGAGCTGTGCTGTTTCGAATGGTTCCAAAACtcaaaggctatt
gataaagctgtatttaatGTTTTAGAGCTGTGCTGTTTCGAATGGTTCCAAAACccaaattttgaagagaaaaaactaaaaac
tGTTTTAGAGCTGTGCTGTTTCGAATGGTTCCAAAACcaaagaactttgtgtctcaatttgtgagctGTTTTAGAGCTGTGCT
GTTTCGAATGGTTCCAAAACatcacttctttcgcttcttcaatattcattGTTTTAGAGCTGTGCTGTTTCGAATGGTTCCAA
AACtcttgataatgaatttgagtcagcaatacaGTTTTAGAGCTGTGCTGTTTCGAATGGTTCCAAAACcgctaaagcttgcc
ctaaagttactgttgaGTTTTAGAGCTGTGCTGTTTCGAATGGTTCCAAAACTagatttctacgaacaagtggattgagaaaG
TTTTAGAGCTGTGCTGTTTCGAATGGTTCCAAAACtcagtcagttaaagacaccgttattaataaGTTTTAGAGCTGTGCTGT
TTCGAATGGTTCCAAAACttgaccttttacgttaacagtccattttgaGTTTTAGAGCTGTGCTGTTTCGAATGGTTCCAAAA
CaacctcattattaaatttcactactactgcGTTTTAGAGCTGTGCTGTTTCGAATGGTTCCAAAACtcttttaaagcagttg
atatcaagacatctGTTTTAGAGCTGTGCTGTTATTATGCTAGGGCACCAttgtggtgttctagtttttttgttatactgaaat
aaattttcagagaatgtgggggaaggcggtaattagattaattcaagatgtaattcagaacttagttggccaagctaacgaaa
tcaccccagtttatcagtttgattgggaaacttatatattggcgactaaaaaatatggacgtcatttagaggtgtgtctattag
tagaaaattcgaattatttttcggattcaaagagaatgtgtcgatagaagagatacgaaagactataattccaaccttatggt
taaagggctaggtgtctacgcttggccaattatttggaacgc

Isolate 537

tttcttgataggcgaatatggtataatattagtaaaagcacagtaataacaaggaatcatcgaaactgaagtcctgctgagac
gaatggcgcgattacgaaagctcaaaagaaaattttctacgagGTTTTAGAGCTGTGCTGTTTCGAATGGTTCCAAAACtgga
attcaaagcaaaagcgcttatgaaatGTTTTAGAGCTGTGCTGTTTCGAATGGTTCCAAAACtgctagacttgaaaaagacag
ttcagacatGTTTTAGAGCTGTGCTGTTTCGAATGGTTCCAAAACattaaaacctcccattatagctctacccgcGTTTTAGA
GCTGTGCTGTTTCGAATGGTTCCAAAACacaacaatattgtcaataagtaccaacccgGTTTTAGAGCTGTGCTGTTTCGAAT
GGTTCCAAAACtaataactaaaaattccacgcttgatttagGTTTTAGAGCTGTGCTGTTTCGAATGGTTCCAAAACtcaaag
gctattgataaagctgtatttaatGTTTTAGAGCTGTGCTGTTTCGAATGGTTCCAAAACccaaattttgaagagaaaaaact
aaaaactGTTTTAGAGCTGTGCTGTTTCGAATGGTTCCAAAACcaaagaactttgtgtctcaatttgtgagctGTTTTAGAGC
TGTGCTGTTTCGAATGGTTCCAAAACatcacttctttcgcttcttcaatattcattGTTTTAGAGCTGTGCTGTTTCGAATGG
TTCCAAAACtcttgataatgaatttgagtcagcaatacaGTTTTAGAGCTGTGCTGTTTCGAATGGTTCCAAAACcgctaaag
cttgccctaaagttactgttgaGTTTTAGAGCTGTGCTGTTTCGAATGGTTCCAAAACtcagtcagttaaagacaccgttatt
aataaGTTTTAGAGCTGTGCTGTTTCGAATGGTTCCAAAACttgaccttttacgttaacagtccattttgaGTTTTAGAGCTG
TGCTGTTTCGAATGGTTCCAAAACaacctcattattaaatttcactactactgcGTTTTAGAGCTGTGCTGTTTCGAATGGTT
CCAAAACtcttttaaagcagttgatatcaagacatctGTTTTAGAGCTGTGCTGTTATTATGCTAGGGCACCAttgtggtgtt
ctagtttttttgttatactgaaataaattttcagagaatgtgggggaaggcggtaattagattaattcaagatgtaattcagaa
cttagttggccaagctaacgaaatcaccccagtttatcagtttgattgggaaacttatatattggcgactaaaaaatatggacg
tcatttagaggtgtgtctattagtagaaaattcgaattatttttcggattcaaagagaatgtgtcgatagaagagatacgaaa
gactataattccaaccttatggttaaagggctaggtgtctacgcttagctaatatt

Isolate 538

gataggcgaatatggtataatattagtaaaagcacagtaataacaaggaatcacccgaaactgaagtcctgctgagacgaatg
gcgcgattacgaaagctcaaaagaaaattttctacgagGTTTTAGAGCTGTGCTGTTTCGAATGGTTCCAAAACtggaattca
aagcaaaagcgcttatgaaatGTTTTAGAGCTGTGCTGTTTCGAATGGTTCCAAAACtgctagacttgaaaaagacagttcag
acatGTTTTAGAGCTGTGCTGTTTCGAATGGTTCCAAAACattaaaacctcccattatagctctacccgcGTTTTAGAGCTGT
GCTGTTTCGAATGGTTCCAAAACacaacaatattgtcaataagtaccaacccgGTTTTAGAGCTGTGCTGTTTCGAATGGTTC
CAAAACtaataactaaaaattccacgcttgatttagGTTTTAGAGCTGTGCTGTTTCGAATGGTTCCAAAACtcaaaggctat
tgataaagctgtatttaatGTTTTAGAGCTGTGCTGTTTCGAATGGTTCCAAAACccaaattttgaagagaaaaaactaaaaa
ctGTTTTAGAGCTGTGCTGTTTCGAATGGTTCCAAAACcaaagaactttgtgtctcaatttgtgagctgttttagagctgtgc
tgtttcgAATGGTTCCAAAACatcacttctttcgcttcttcaatattcattGTTTTAGAGCTGTGCTGTTTCGAATGGTTCCA

AAACTcttgataatgaatttgagtcagcaatacaGTTTTAGAGCTGTGCTGTTTCGAATGGTTCCAAAACcgctaaagcttgc
cctaaagttactgttgaGTTTTAGAGCTGTGCTGTTTCGAATGGTTCCAAAACtcagtcagttaaagacaccgttattaataa
GTTTTAGAGCTGTGCTGTTTCGAATGGTTCCAAAACttgaccttttacgttaacagtccattttgaGTTTTAGAGCTGTGCTG
TTTCGAATGGTTCCAAAACaacctcattattaaatttcactactactgcGTTTTAGAGCTGTGCTGTTTCGAATGGTTCCAAA
ACtcttttaaagcagttgatatcaagacatctGTTTTAGAGCTGTGCTGTTATTATGCTAGGGCACCAttgtggtgttctagt
tttttgttatactgaaataaattttcagagaatgtgggggaaggcggtaattagattaattcaagatgtaattcagaacttag
ttggccaagctaacgaaatcaccccagtttatcagtttgattggaaacttatatattggcgactaaaaaatatggacgtcatt
tagaggtgtgtctattagtagaaaattcgaattatttttcggattcaaagagaatgtgtcgatagaagagatacgaaagacta
taattccaaccttatggttaaagggctaggtgtctacgc
